# Refactoring the architecture of a polyketide gene cluster enhances docosahexaenoic acid production in *Yarrowia lipolytica* through improved expression and genetic stability

**DOI:** 10.1186/s12934-023-02209-9

**Published:** 2023-09-29

**Authors:** Demian Dietrich, Sofija Jovanovic-Gasovic, Peng Cao, Michael Kohlstedt, Christoph Wittmann

**Affiliations:** Institute of Systems Biotechnology, Saarland University, Saarbrücken, Germany

**Keywords:** *Yarrowia lipolytica*, PUFA, DHA, Metabolome, Transcription, Acetyl-CoA, Malonyl-CoA, Genetic stability, Glycerol, Synthetic cluster, Synthetic biology, Transcriptome

## Abstract

**Background:**

Long-chain polyunsaturated fatty acids (LC-PUFAs), such as docosahexaenoic acid (DHA), are essential for human health and have been widely used in the food and pharmaceutical industries. However, the limited availability of natural sources, such as oily fish, has led to the pursuit of microbial production as a promising alternative. *Yarrowia lipolytica* can produce various PUFAs via genetic modification. A recent study upgraded *Y. lipolytica* for DHA production by expressing a four-gene cluster encoding a myxobacterial PKS-like PUFA synthase, reducing the demand for redox power. However, the genetic architecture of gene expression in *Y. lipolytica* is complex and involves various control elements, offering space for additional improvement of DHA production. This study was designed to optimize the expression of the PUFA cluster using a modular cloning approach.

**Results:**

Expression of the monocistronic cluster with each gene under the control of the constitutive *TEF* promoter led to low-level DHA production. By using the *minLEU2* promoter instead and incorporating additional upstream activating UAS1B4 sequences, 5' promoter introns, and intergenic spacers, DHA production was increased by 16-fold. The producers remained stable over 185 h of cultivation. Beneficially, the different genetic control elements acted synergistically: UAS1B elements generally increased expression, while the intron caused gene-specific effects. Mutants with UAS1B16 sequences within 2–8 kb distance, however, were found to be genetically unstable, which limited production performance over time, suggesting the avoidance of long repetitive sequence blocks in synthetic multigene clusters and careful monitoring of genetic stability in producing strains.

**Conclusions:**

Overall, the results demonstrate the effectiveness of synthetic heterologous gene clusters to drive DHA production in *Y. lipolytica*. The combinatorial exploration of different genetic control elements allowed the optimization of DHA production. These findings have important implications for developing *Y. lipolytica* strains for the industrial-scale production of valuable polyunsaturated fatty acids.

**Supplementary Information:**

The online version contains supplementary material available at 10.1186/s12934-023-02209-9.

## Background

Long-chain polyunsaturated fatty acids (LC-PUFAs) exhibit a variety of health benefits that have enabled their application in the food and pharmaceutical industries [1]. One of the most prominent LC-PUFAs is docosahexaenoic acid (DHA, 22: 6, ω3). It functions as an essential nutrient throughout the lifespan and supports brain and retina development and function, among other functions [2]. Humans lack sufficient capability to derive DHA from endogenous α-linolenic acid (ALA, 18: 3, ω3), making this PUFA a crucial part of our diet [3, 4]. Health organizations worldwide recommend the daily intake of DHA [2, 5]. Oily fish, however, the natural source of DHA, are becoming increasingly limited due to the increasing depletion of wildlife ocean populations caused by overfishing and to constantly declining DHA levels in hatched fish, resulting from decreased DHA levels in phytoplankton due to global warming, which translates through all species up the food chain [6]. One of the most promising alternatives to derive sustainable DHA lies in microbial production, with the oleaginous yeast *Yarrowia lipolytica* as one of the major production hosts [5, 7]. This microbe has been used in industry for 70 years [8], has proven feasibility for genetic modification [9–11], and allows the safe manufacturing of GRAS-awarded products [12], including organic acids [13], biofuels [14], enzymes [15], and high-value chemicals for nutritional and pharmaceutical applications [16].

**Fig. 1:**
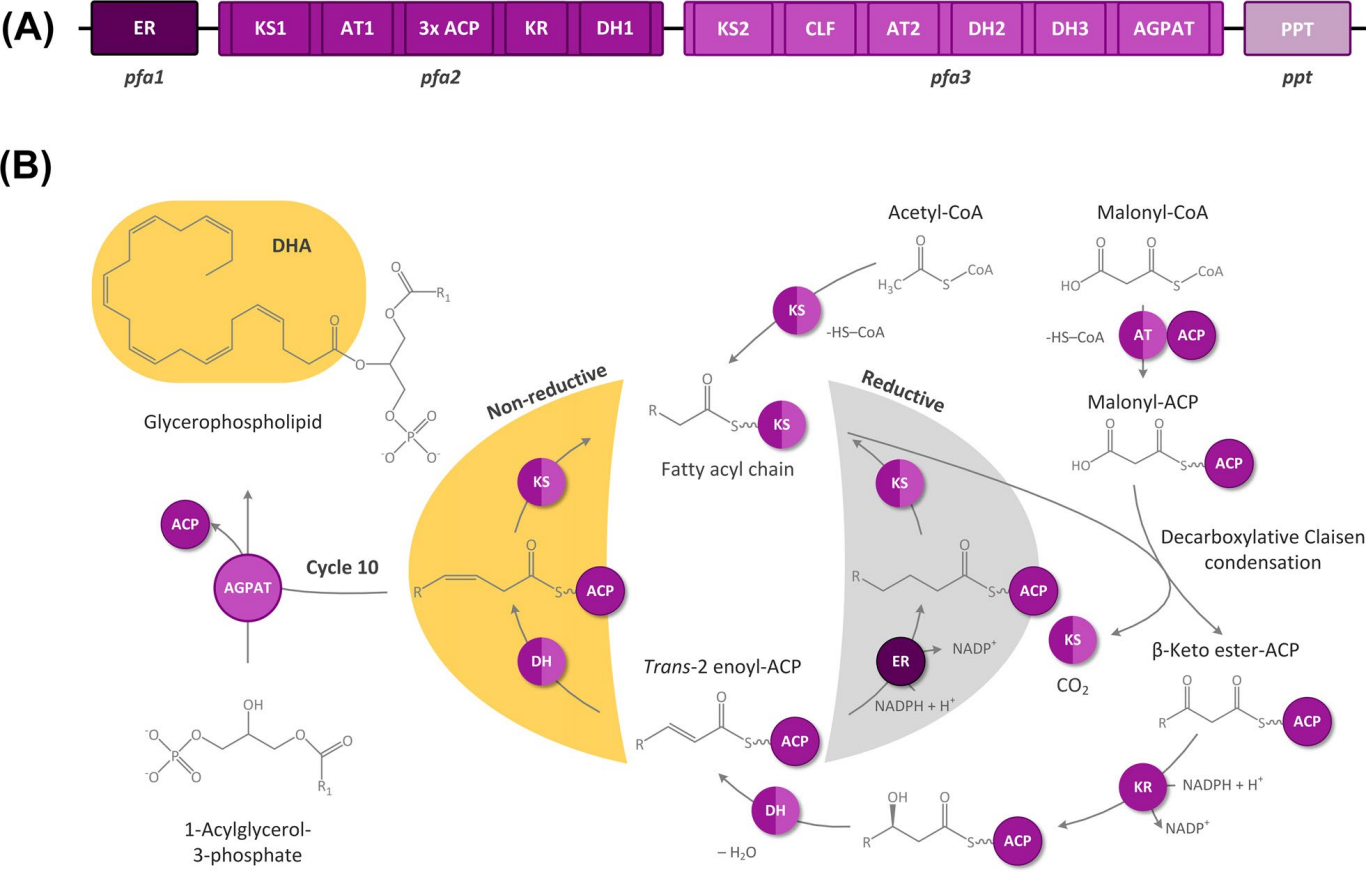
Genetic and metabolic design to produce docosahexaenoic acid (DHA) in *Yarrowia lipolytica* through heterologous expression of a myxobacterial PKS-like PUFA synthase gene cluster. The PUFA cluster from *Aetherobacter fasciculatus* (SBSr002) comprised the three genes *pfa123*, encoding the multidomain subunits of the PUFA synthase, as well as *ppt*, encoding 4'-phosphopantetheinyl transferase (PPTase) (**A**). The biosynthesis of DHA by PUFA synthase starts with acetyl-CoA, which binds to the ketosynthase domain and is then successively elongated with activated malonyl-CoA units bound to an acyl carrier protein (ACP) (**B**). Within each cycle, decarboxylative Claisen condensations form β-keto ester intermediates, which are then reduced to the corresponding alcohols. Then, dehydration inserts an α, β-*trans* double bond, which is either reduced or isomerized into the *cis* form. After ten cycles, the formed DHA fatty acyl chain is transferred to the 2-position of 1-acylglycerol-3-phosphate by 1-acylglycerol-3-phosphate O-acyltransferase (AGPAT)

In an advantage for PUFA production, *Y. lipolytica* naturally accumulates high amounts of lipids, and this potential has been exploited to overproduce ALA, γ-linolenic acid (GLA, 18: 3, ω6) and eicosapentaenoic acid (EPA, 20:5, ω3) by extending the yeast’s native fatty acid biosynthesis via heterologous elongases and desaturases [17–19]. Using an alternative strategy, *Y. lipolytica* was recently streamlined for DHA production [20]. The expression of a four-gene cluster from the myxobacterium *Aetherobacter fasciculatus* (SBSr002), encoding a polyketide-like synthase [21], allowed DHA to be synthetized independently of native fatty acids in the recombinant strain *Y. lipolytica* Af4, allowing a significantly reduced demand for redox power (Fig. 1) [20]. The PUFA cluster was expressed from a well-suited integration site [20], among various options previously evaluated for heterologous gene expression in *Y. lipolytica* [22, 23]. The cluster itself was only used in the form of a single genetic layout that expressed each of the four genes under an individual promoter. It is, however, well known that the expression of genes in *Y. lipolytica* is based on various control elements, such as distal (UAS1) and proximal (UAS2) upstream activating enhancers, promoters, terminators, and intron sequences. Beyond the previous work, the combinatorial use of such elements offered additional space to improve DHA production and expand our presently limited knowledge on how to best express myxobacterial genes in yeast [24–26].

Therefore, we explored various alternative designs to overexpress the PUFA cluster in *Y. lipolytica*. In an initial test round, different core promoters, upstream activating enhancers, terminators, and introns were systematically combined, which yielded a set of PUFA clusters that were then genomically integrated into yeast. The obtained mutants were evaluated for performance, which revealed a strong impact of the cluster design on production efficiency. In addition, multiomics analysis elucidated several features of the strains, including (i) genetic stability, (ii) individual expression levels of the PUFA genes, and (iii) intracellular availability of the DHA precursors acetyl-CoA and malonyl-CoA. Further optimization cycles based on this new understanding iteratively balanced and optimized the expression of the individual genes in the cluster. The best strain, *Y. lipolytica U4 minLEU2 INT S* Af4, expressed the cluster with compact upstream activating sequences (U4), 5^’^-introns (INT), and spacers (S) under the control of the *minLeu2* promoter. The mutant achieved a 16-fold increase in DHA content to 17.1% of total fatty acids compared to a basic strain mutant that expressed a minimal cluster under the control of a strong constitutive *TEF* promoter.

## Results

### Heterologous expression of a myxobacterial PUFA cluster under control of the constitutive TEF promoter enables low-level DHA production

**Fig. 2:**
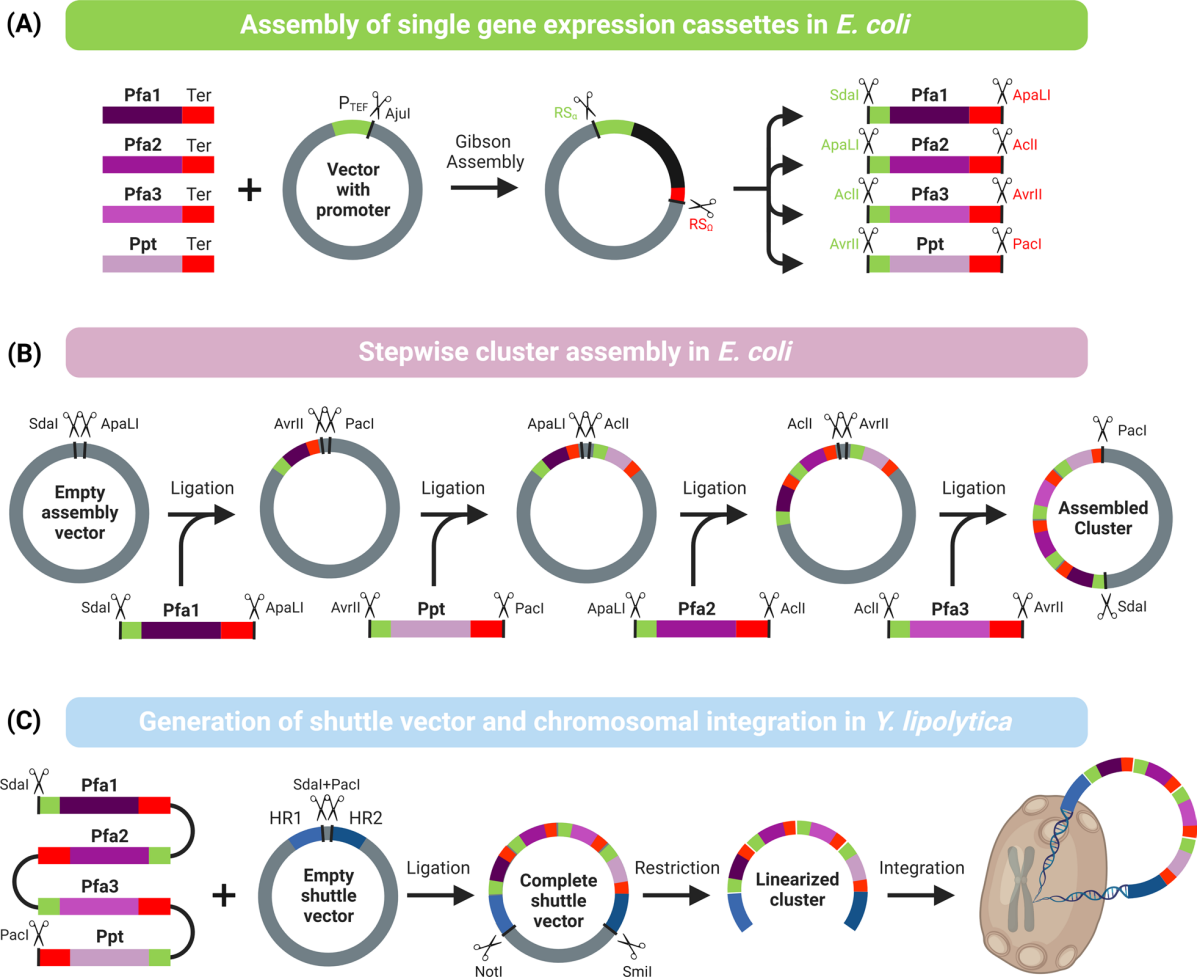
Modular workflow for the genetic assembly of multigene clusters for genomic expression in *Yarrowia lipolytica*. The use of specific restriction enzymes enables the correct modular assembly of repetitive single gene modules into four-gene PUFA synthase gene clusters. First, single PUFA cluster genes are fused with a terminator. The resulting DNA fragments are integrated into a preconfigured promoter vector using Gibson assembly. The promoter-gene-terminator elements are excised from the vector using restriction digestion with AjuI (**A**) and subsequently integrated into an assembly vector in a defined order using unique restriction sites (**B**), which yields the complete cluster. The cluster is inserted into a shuttle vector between two homology domains using restriction and ligation. The cluster is then excised for chromosomal integration into *Y. lipolytica* (**C**). The figure was created using Biorender.com

**Fig. 3:**
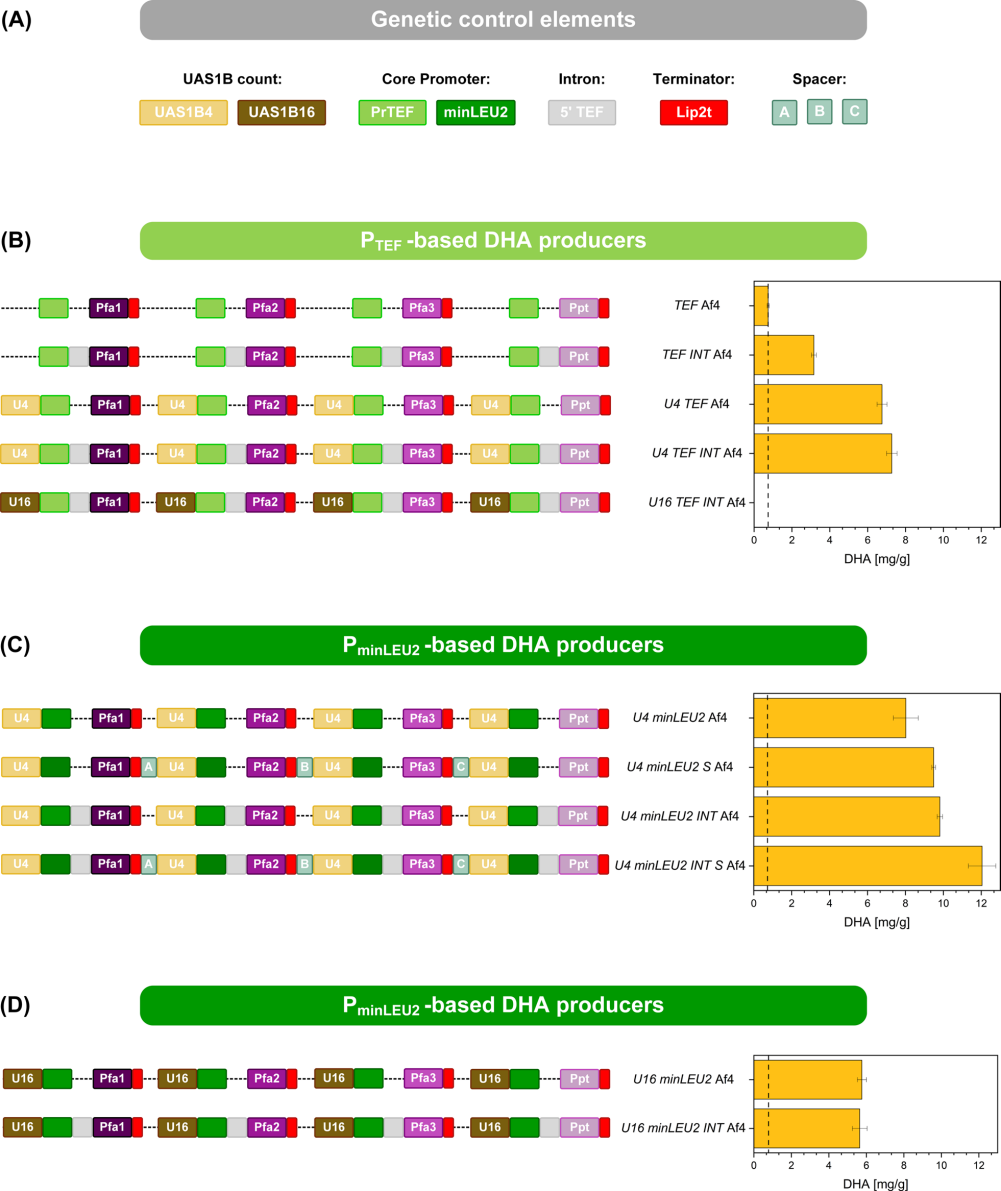
Metabolic engineering of *Yarrowia lipolytica* to produce docosahexaenoic acid (DHA) via differently designed heterologous PUFA gene clusters. The cluster designs were based on different combinations of genetic control elements to express each of the four cluster genes (*pfa1*, *pfa2*, *pfa3*, *ppt*), including UAS1B element blocks of different lengths, the two core promoters *pTEF* and *pminLEU2*, the 5ʹ *TEF* intron, 200 bp spacers, and the Lip2t terminator (**A**). The created strains were evaluated for production efficiency in glycerol-grown batch cultures, whereby the DHA level (mg g_CDM_^−1^) was measured after 185 h (**B, C, D**). The data show the genetic layout and performance of first-generation strains based on the *TEF* promoter (**B**) and second-generation strains based on the *minLEU2* promoter (**C**, **D**). n = 3

In pursuit of recombinant DHA overproduction in *Y. lipolytica*, we aimed to optimize the expression of a myxobacterial four-gene cluster encoding a polyketide-like PUFA synthase [21]. To streamline the experimental workflow for constructing different cluster variants, we set up a modular approach, based on a set of specific restriction enzymes to create unique cleavage sites during cloning (SmaI, SdaI, ApaLI, AclI, AvrlI, PacI, AjuI, NotI, SmiI). This strategy enabled a straightforward combinatorial assembly of the cluster genes with different genetic control elements of interest in the correct order (Figs. 2, 3A). It also allowed easy replacement of individual sequence elements during the later optimization.

Initially, we designed a basic cluster that expressed each of the four PUFA genes (*pfa1, pfa2, pfa3, ppt*) under the control of the well-characterized constitutive *TEF* promoter (*P*_*TEF*_). In the first step of construction, individual gene-terminator fusion constructs were integrated into plasmids that carried the *P*_*TEF*_ promoter upstream of the integration site. The plasmids were amplified in *E. coli* and used to obtain the corresponding promoter-gene-terminator cassettes (Fig. 2A).

The four single-gene cassettes were then cloned stepwise into an assembly vector using restriction digestion, which provided the entire synthetic 19.1 kb PUFA cluster in the vector (Fig. 2B). The cluster sequence was excised and ligated into a shuttle vector. Finally, this provided the linearized cluster flanked by the two homology domains (Fig. 2C).

**Fig. 4:**
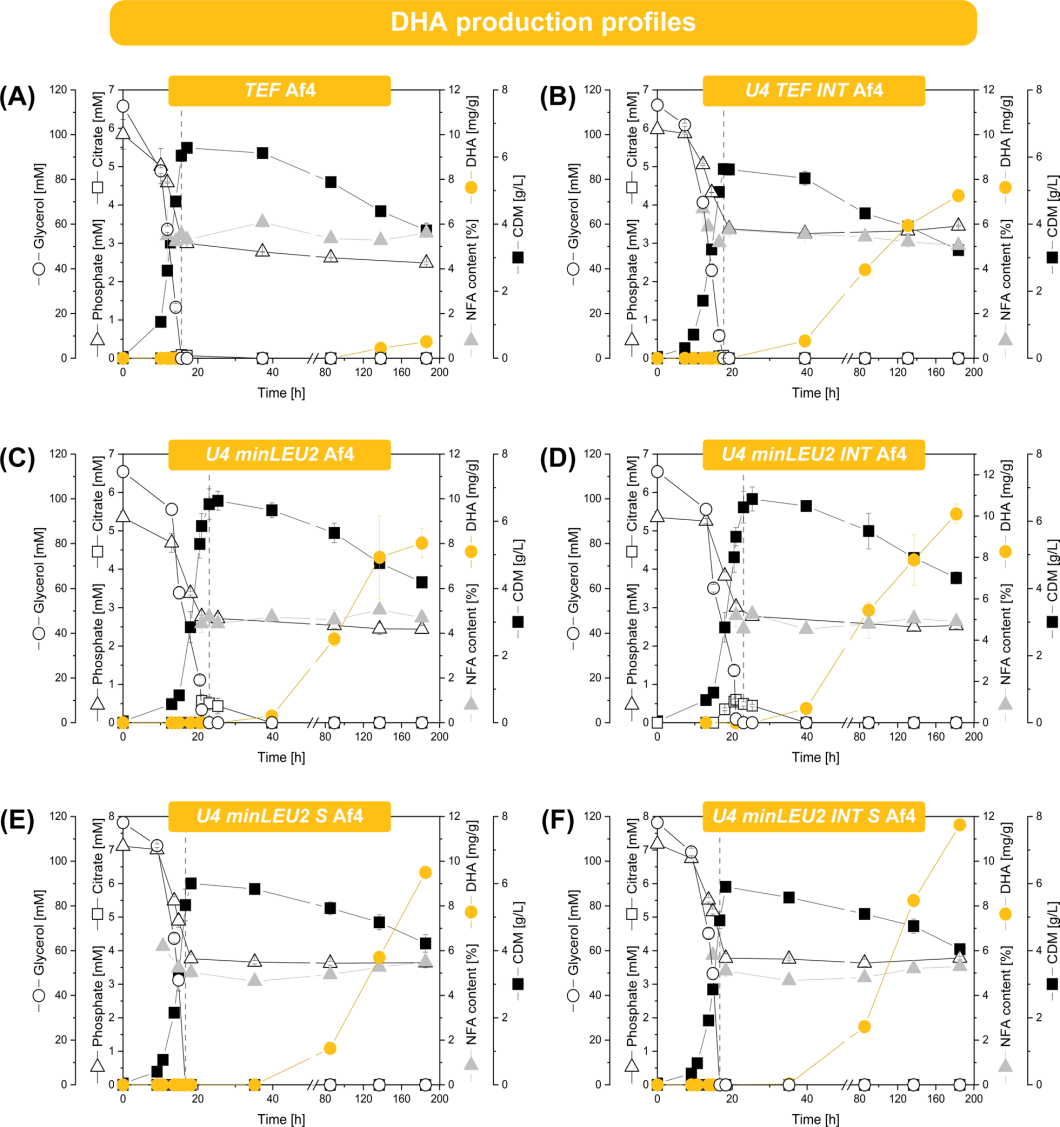
Impact of the genetic architecture of heterologous PUFA clusters on the production of docosahexaenoic acid (DHA) in recombinant *Y. lipolytica*. The strains differed in the genetic architecture of the DHA cluster as follows: *TEF* Af4 (**A**), *U4 TEF INT* Af4 (**B**), *U4 minLEU2* Af4 (**C**), *U4* *minLEU2INT* Af4 (**D**), *U4* *minLEU2* *S* Af4 (**E**), and *U4* *minLEU2* *INT* *S* Af4 (**F**).  They were cultivated in glycerol-based minimal medium. The time point of glycerol depletion is indicated by a dotted line. The different genetic cluster layouts are shown in Fig. 3. NFA = native fatty acids. n = 3

Genomic integration of the cluster into the nonproducing host *Y. lipolytica* Po1h yielded strain *Y. lipolytica TEF* Af4. When grown on a glycerol-based minimal medium, the mutant formed 0.8 mg DHA g^−1^ h^−1^, constituting 1.3% of total fatty acids (Fig. 3B). The strain consumed all glycerol during an initial phase of exponential growth (µ = 0.34 h^−1^) and reached a maximum concentration of cell dry mass (CDM) of 6.3 g L^−1^ (Fig. 4A). Citrate, an often-observed overflow metabolite of the yeast, was formed only in traces (< 1 mM). DHA was only detectable after 85 h. The parent nonproducing host *Y. lipolytica* Po1h did not form DHA (as expected) but otherwise revealed similar growth behaviour (data not shown). In summary, a lean Af4 cluster design was sufficient to enable DHA formation in *Y. lipolytica*. However, only a small amount of DHA was formed, and unexpectedly, the product accumulated only during the late stationary phase, despite the presumably constitutive nature of the promoter used [27].

### The implementation of additional upstream activating sequences and 5’ promoter introns improved the formation of DHA up to tenfold

In pursuit of improved production, we created four *P*_*TEF*_*-*based variants. In comparison to the basic strain *TEF* Af4, the second-generation mutants comprised different sets of additional genetic control elements upstream and downstream of the *TEF* promoter. The established workflow proved efficient, as the new design could be quickly, easily, and precisely realized. The incorporation of the 5’ *TEF* intron between the promoter and the corresponding gene resulted in strain *Y. lipolytica TEF INT* Af4*,* which, encouragingly, revealed four-fold increased DHA production (Fig. 3B). The implementation of blocks of four activating UAS1B elements upstream of each promoter resulted in the production of nine-fold more DHA in *Y. lipolytica U4 TEF* Af4, compared to the parent strain. *Y. lipolytica U4 TEF INT* Af4*,* exhibiting both modifications, achieved an even higher DHA content of 7.3 mg g_CDM_^−1^ (Fig. 3B). Favourably, the two modifications acted synergistically. It was interesting to note that strain *U4 TEF INT* Af4 visibly formed DHA after 40 h and that the cells maintained high productivity until the end of the process, while the overall growth behaviour remained relatively unaffected.

We hypothesized that more than four *UAS1B* copies might result in even stronger gene expression, because the elements were obviously beneficial for DHA overproduction (Fig. 3B). Therefore, we constructed an extended 26.3 kb cluster variant that comprised blocks of sixteen UAS1B elements in front of each *P*_*TEF*_. The synthetized and assembled cluster was genomically integrated into the wild type, yielding strain *U16 TEF INT* Af4. Contrary to expectations, however, this version did not produce any DHA (Fig. 3B) but was otherwise unaffected in substrate use, growth, and biomass formation (Additional file 1: Figure S1C).

In summary, all *P*_*TEF*_-based strains (except the one with sixteen UAS1B repeats) enabled DHA production. The genetic architecture of the heterologous PUFA cluster influenced the achieved DHA level, indicating that the production performance was transcriptionally limited. All *P*_*TEF*_-based strains exhibited similar characteristics regarding the use of glycerol and phosphate as well as growth. Therefore, neither the expression of the PUFA cluster nor the accumulation of DHA seemed to have an impact on the gross metabolic behaviour.

### The use of the stationary-phase minimal LEU2 promoter for expression of the cluster enables a 20% increase in the cellular DHA content

As shown above, the recombinant strains formed DHA mainly during the stationary phase (Fig. 4). We therefore included the minimal *minLEU2* promoter (*P*_*minLEU2*_) for further development [28]. We constructed a set of third-generation strains following the established modular workflow. For comparison, we included different numbers (4 and 16) of UAS1B tandem repeats. Two genomic mutants were generated, namely, *Y.* *lipolytica U4 minLEU2* Af4 and *U16 minLEU2* Af4. After validation by PCR and sequencing, the two strains were evaluated.

The variant with four UAS1B tandem copies accumulated 8 mg g_CDM_^−1^ DHA (Fig. 3C, 4C,), significantly more than the corresponding *P*_*TEF*_ counterpart. Strain *U16 minLEU2* Af4 was less efficient (Fig. 3D, Additional file 1: Figure S1D). We focused on the U4 strain for a fourth optimization round. The additional insertion of *TEF* introns at the 5ʹ end of each gene increased DHA production to 10 mg g_CDM_^−1^ in the mutant *Y. lipolytica U4 minLEU2 INT* Af4, almost 25% more than in the strain without the intron (Fig. 4D, Fig. 3C). As an alternative, we placed 200 bp spacer sequences between each terminator and promoter sequence to reduce the competition for protein binding [29]. This change also improved DHA production (Fig. 3C). Finally, we combined introns and spacers to construct the fifth-generation strain *Y. lipolytica U4 minLEU2 INT S* Af4 (Fig. 3C). When evaluated in the production setup, it achieved 12 mg DHA g_CDM_^−1^ (Fig. 4F), the highest value among all constructed producers. In this regard, the best setup enabled 16-fold better production than the P_*TEF*_-based design used initially.

### Multiple UAS1B repeats destabilize the PUFA cluster and cause the appearance of nonproducing mutants during the production process

**Fig. 5:**
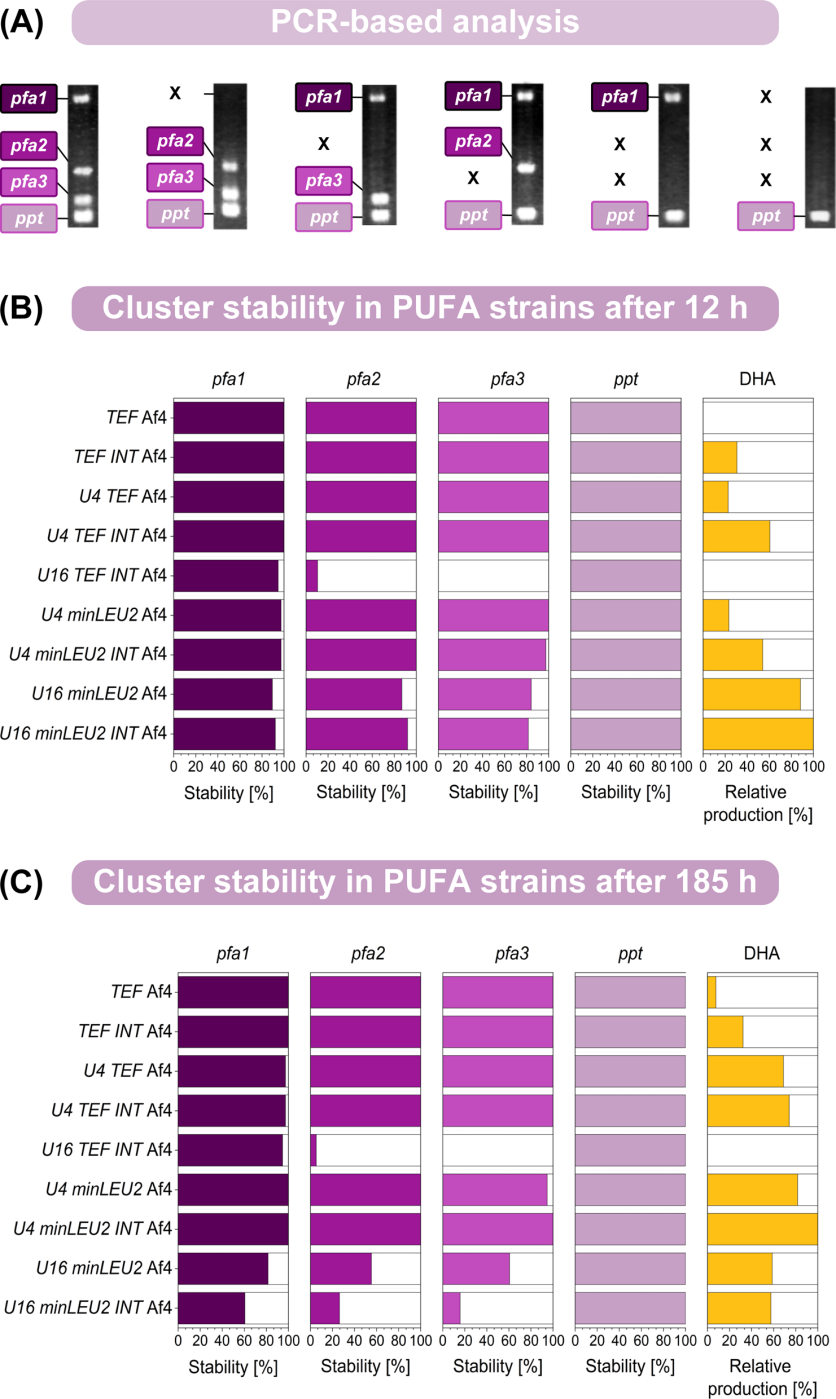
Evaluation of the genetic stability of heterologous PUFA clusters expressed in recombinant *Y. lipolytica* to produce docosahexaenoic acid (DHA). The different genetic cluster layouts can be taken from Fig. 3. The presence of the four cluster genes, *pfa1, pfa2, pfa3, and ppt*, was analysed by colony PCR (**A**). The use of four specific primer pairs resulted in PCR products with clearly distinguishable lengths: 1.72 kb (pfa1), 0.91 kb (pfa2), 0.73 kb (pfa3), and 0.61 kb (ppt), enabling simultaneous analysis. For each strain, we analysed the genetic configuration of 40 clones from cell populations sampled from the production process after 12 h (**B**, early phase) and 185 h (**C**, late phase). The DHA production efficiency for the two phases was estimated after 40 h (early phase) and 185 h (late phase). The relative DHA production is given in comparison to the best strain during each phase, set to 100%. n = 3

As shown, strains with 16 UAS1B elements upstream of each of the four cluster genes failed to efficiently produce DHA, regardless of the promoter used (Fig. 3). Interestingly, two of these multi-UAS1B strains showed superior production performance during the early stages of the process before fading out (Additional file 1: Figures S1D, S1E). Genetic instability was one of the possible reasons for this behaviour. We therefore screened for the stability of the PUFA cluster during production. Because sequencing of the cluster was challenging due to the highly repetitive sequences, we used PCR to determine the presence of each heterologous gene instead (Fig. 5A). The PCR-based approach had been established in synthetic biology work (see above) to verify the presence of the intact cluster in newly generated mutants prior to inoculating them for cryo-preservation. Here, we applied it to screen for cluster stability during production, i.e., during the exponential phase (12 h) and during the late stationary phase (185 h). Altogether, cells were grown for approximately two weeks in each setup, including a two-day preincubated culture for inoculation. For each strain, 40 clones were analysed (Fig. 5, Table 1).

**Table 1:**
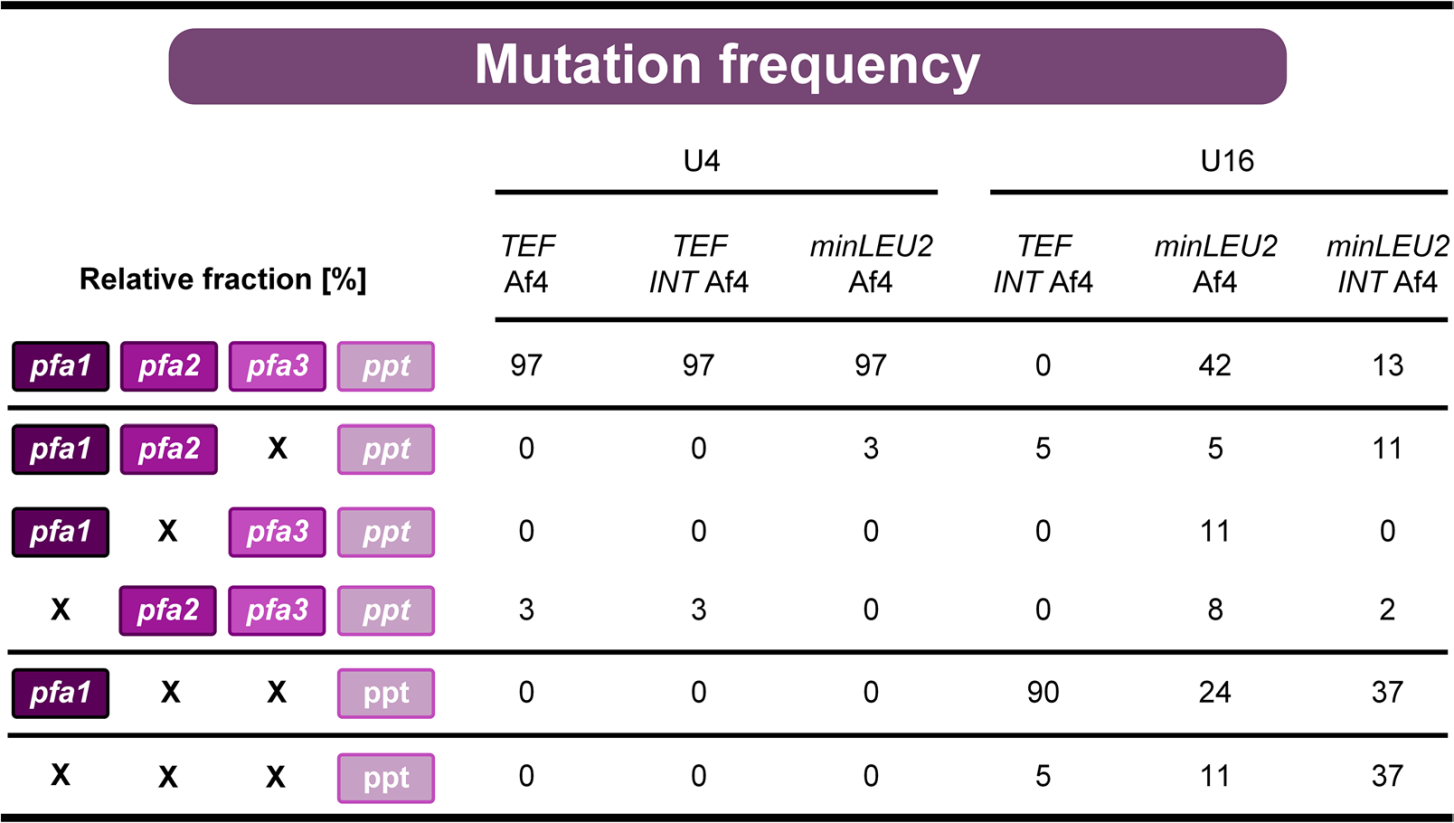
Genetic stability of different synthetic heterologous PUFA clusters during production of the omega-3 fatty acid DHA in recombinant *Y. lipolytica*

The cluster variants were based on different combinations of promoters, upstream activating sequence elements, introns, spacers, and terminators (Fig. 3). The cell populations were analysed for the presence of the four cluster genes *pfa1, pfa2, pfa3,* and *ppt* after culturing for two weeks. For each strain, 40 clones were analysed using PCR, and a set of eight specific primers allowed us to test for the presence of all genes simultaneously. The obtained patterns provided the cluster configuration for each clone and population-based insight into the frequency of different mutation events, which are given as relative fractions in percent

The analysis revealed that higher UAS1B copy numbers caused genetic instability, explaining the significant decrease in production performance. Strains without UAS1B tandem repeats were found to be completely stable over the entire cultivation period (Fig. 5BC). Mutants with four UAS1B tandem repeats in front of each gene remained largely intact (Fig. 5B, 5C). A few clones exhibited the loss of one of the genes during the production process, namely, *pfa1* or *pfa3*. These mutation events, however, were rare, and this picture did not change in related strains that were based on another promoter and an additional intron. Approximately 98% of the tested clones were still intact at the end of the process. However, the findings revealed the occurrence of undesired recombination events within UAS1B4-based clusters.

UAS1B16-based gene clusters were found to be structurally unstable (Fig. 5B, C). The two mutants *Y. lipolytica U16 minLEU2* Af4 and *U16 minLEU2 INT* Af4 gradually lost parts of the cluster during fermentation. After 12 h, up to 90% of the tested clones still contained all cluster genes (Fig. 5B). At the end of the process, however, only 60% of the *U16 minLEU2* Af4 clones were found to be intact. The population of *U16 minLEU2 INT* Af4 comprised only 20% of genetically correct cells during the early phase of production, indicating that the additional intron sequence further decreased stability. The population of strain *U16 TEF INT* Af4 had already largely lost the genes *pfa2* and *pfa3* at the beginning of the production process. Obviously, this was the reason for the lack of DHA production observed for this mutant. It appeared that the strain had already decomposed during preculturing. In summary, strains with four UAS1B elements appeared optimal, as they offered high stability together with high production efficiency. The stability of the *ppt* gene was not affected in any of the mutants, because it was outside the repetitive sequence blocks. The two genes in the middle exhibited the highest instability, likely due to the possibility of recombination events in both directions.

### Four-copy UAS1B tandem repeats enable generally increased cluster expression

**Fig. 6:**
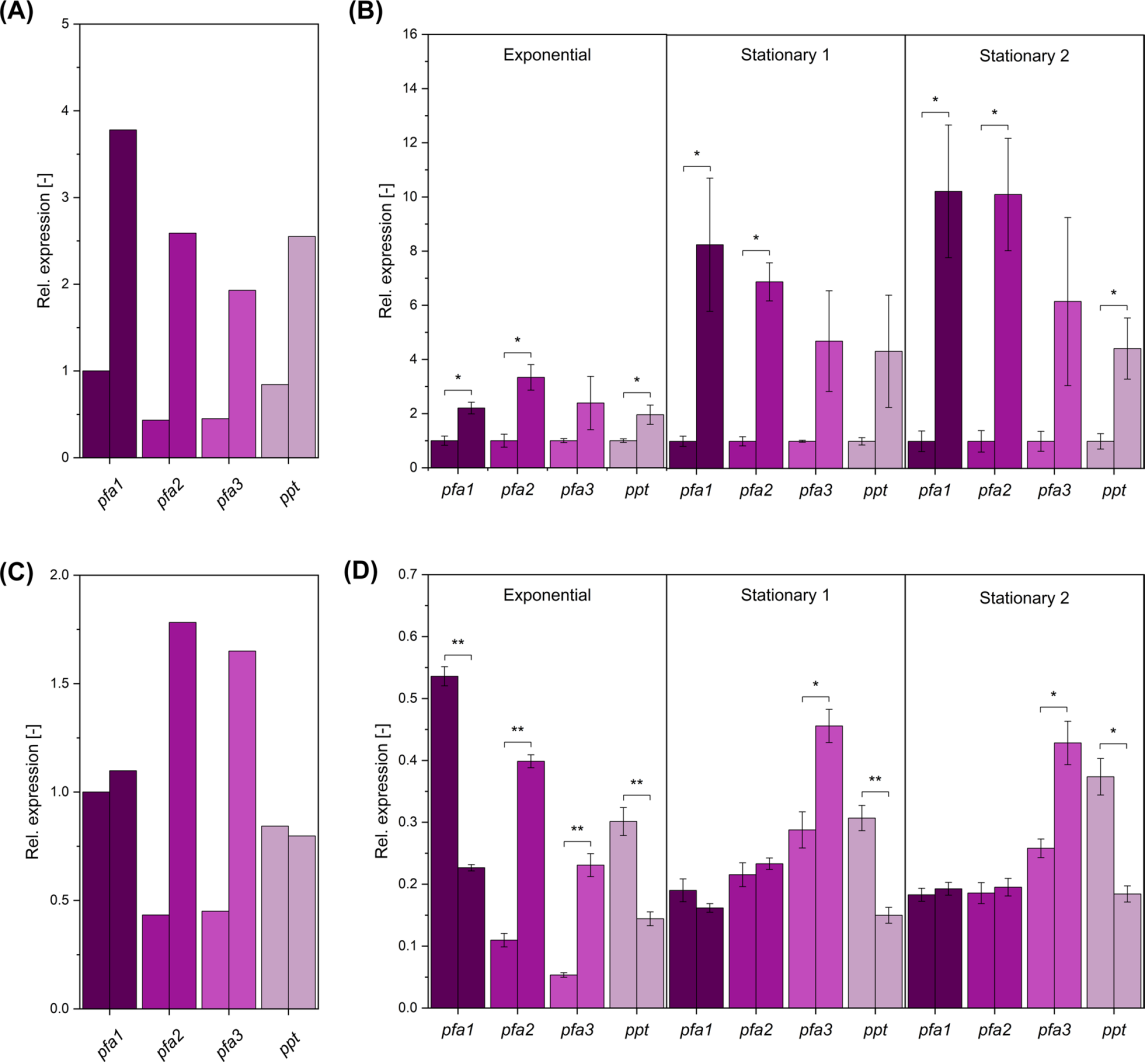
Impact of upstream enhancing UAS1B elements (U4) and the *TEF* intron (INT) on the expression strength of the PUFA cluster genes *pfa1, pfa2, pfa3,* and *ppt* during glycerol-based docosahexaenoic acid (DHA) production in *Y. lipolytica*. The data comprise the average (**A**) and the time-resolved gene expression (**B**) in the strains *TEF* Af4 (left bars) and U4 *TEF* Af4 (right bars). For the latter, the time-resolved expression was measured during the exponential phase (ca. 15 h), the early production phase (stationary 1, ca. 38 h), and the late production phase (stationary 2, ca. 135 h). The same data are shown for the strains *TEF* Af4 (left bars) and *TEF INT* Af4 (right bars) (**C, D**). The gene expression level was measured using q-RT‒PCR. Statistical significance was assessed by Student’s t test (* p = 0.05 and ** p = 0.01). n = 3

To link the observed production differences to the expression levels of the cluster genes, we examined the obtained mutants during the exponential growth phase, the early production phase, and the late production phase using q-RT‒PCR. Although the observed patterns were generally complex, several significant findings could be extracted. As exemplified for strains *TEF* Af4 and *U4 TEF* Af4, the use of the UAS1B elements increased the expression of all cluster genes independently of the time point during fermentation (Fig. 6A). The expression increase was strongest during the early and late stationary phases and resulted in up to tenfold higher expression levels for the genes *pfa1* and *pfa2* (Fig. 6B). In this regard, the use of the activating sequence motifs shifted the expression of the clusters towards the stationary phase, when most of the DHA was produced. Similar enhancing effects were also observed for other mutants (Additional file 1: Figure S3A).

Furthermore, interesting effects were caused by the insertion of the TEF intron between the promoter and cluster gene. When used in combination with P_TEF_, the intron resulted in time- and gene-dependent alterations in the expression level. It preferentially increased the expression of *pfa2* and *pfa3*, the two inner cluster genes, while decreasing the expression of the two flanking genes *pfa1* and *ppt*. For *pfa3* and *ppt*, stimulating and attenuating effects, respectively, were observed in all culture phases. For *pfa1* and *pfa*, however, an effect was observed only for the exponential phase (when DHA was not produced). On average, normalized to the expression level of *pfa1* (set to 1), the use of the intron resulted in a pronounced rebalancing of gene expression (Fig. 6C, D). The two inner genes, which were expressed approximately twofold less than the two outer genes in strain TEF Af4, were strongly upregulated by the intron. This resulted in an almost fourfold higher expression ratio between the inner and outer genes. Interestingly, the use of the TEF intron did not cause similarly significant changes in strains that were based on *P*_*minLEU2*_, ultimately due to an incompatibility of the naturally noncooperating genetic elements (Additional file 1: Figures S3C, S3D).

### Enhanced DHA biosynthesis results in the depletion of the intracellular acetyl-CoA and malonyl-CoA pools

**Fig. 7:**
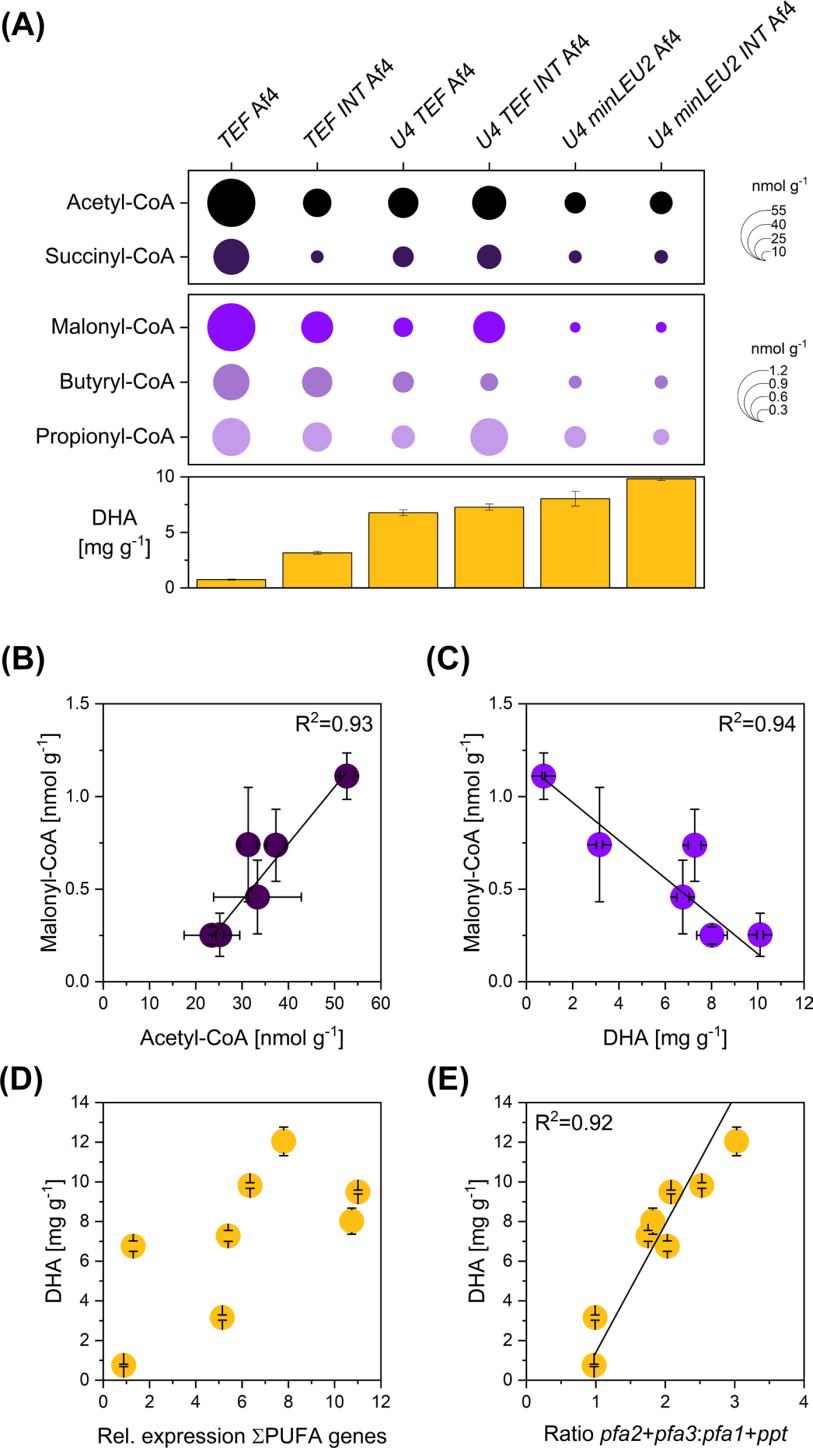
Multiomics view of the DHA-producing *Y. lipolytica* strain genealogy. The absolute levels of intracellular CoA thioesters were determined using LC‒MS/MS and internal ^13^C standards (**A**). Samples were taken during the early production phase (20 h after the maximum biomass concentration had been reached). The data were used to inspect the relation between the intracellular levels of acetyl-CoA and malonyl-CoA, the two precursors for DHA biosynthesis (**B**), and the intracellular levels of malonyl-CoA and DHA production (**C**). In addition, gene expression data in the different producers are shown in relation to DHA production (**D, E**). Here, the total expression strength, i.e., the sum of the average expression of all four cluster genes during the DHA production phase (**D**), as well as the expression ratio between *pfa2* + *pfa3* versus *pfa1* + *ppt* (**E**) during the DHA production phase, are given. The values represent the average expression after 38 and 135 h. n = 3

As an efficient precursor supply was known from previous work to be crucial for metabolite overproduction [30–33], we were interested in determining which levels of production affected the availability of the two CoA-thioester pools among the created strain genealogy. We therefore quantified the absolute levels of several CoA-thioesters during DHA production using LC‒MS/MS and internal ^13^C-labelled standards (Fig. 7). Acetyl-CoA was the most abundant CoA ester, followed by succinyl-CoA. In comparison, malonyl-CoA, propionyl-CoA, and butyryl-CoA were present at 10–50-fold lower levels. The six studied producers differed strongly in the CoA-thioester spectrum (Fig. 7A). Interestingly, the intracellular CoA-ester levels closely correlated with DHA production. For example, the best producer contained 50% less acetyl-CoA than the producer with the lowest efficiency. The pool of malonyl-CoA showed an even stronger effect. The best producer contained 80% less of the precursor than the one with the lowest DHA titre. Similar trends were observed for other CoA esters, suggesting that the different pools were, to some extent, actively equilibrated. This effect was obvious for the pools of acetyl-CoA and malonyl-CoA, which appeared tightly coupled among all producers (R^2^ = 0.93) (Fig. 7B). A linear correlation was also obtained between DHA production and intracellular malonyl-CoA availability (R^2^ = 0.94), revealing that the engineered producers were not able to fully replenish the precursor pool during product synthesis (Fig. 7C), which indicated a potential bottleneck in precursor supply that became increasingly prominent with stepwise strain improvement. On the transcriptional side, mean gene expression showed a positive trend towards higher expression (Fig. 7D). Digging deeper into the link between transcription and production performance, the inspection of expression ratios between different genes of the cluster revealed another interesting insight. Across all strains, an appropriate ratio between the expression of the genes *pfa2* plus *pfa3* to the genes *pfa1* plus *ppt*, i.e., between the inner and the outer cluster genes, seemed to be crucial for high DHA production (Fig. 7E).

## Discussion

### A streamlined promoter architecture of a myxobacterial PUFA gene cluster improves DHA production in recombinant Y. lipolytica

The promoter architecture has great potential to balance transcription in the oleaginous yeast *Y. lipolytica* [26]. For example, the expression of a two-gene pathway as one operon, as a pseudo-operon, and in a monocistronic layout resulted in significantly different expression levels [27]. So far, transcriptional responses are difficult to predict, suggesting systematic testing and fine-tuning of different genetic layouts for a given problem [27]. In this work, we aimed to streamline the architecture of a myxobacterial four-gene cluster (Fig. 1) that encoded a PKS-like DHA assembly line to drive the production of DHA (C22: 6, ω-3), an omega-3 fatty acid with multiple benefits for human health and substantial commercial value [34].

As shown, we created a spectrum of novel producers by combinatorically exploring a set of regulatory control elements, including the promoters *P*_*TEF*_ [35] and *P*_*minLEU2*_ [20], upstream activating sequence blocks (UAS1B) of different lengths [36], introns [32], and intergenic spacers. We generally designed the different cluster variants in monocistronic form. Each gene was expressed from its own promoter and separated from upstream transcription by a terminator. This architecture was chosen to avoid transcriptional inhibition effects that could have otherwise resulted in multigene expression from one promoter [37] and nonfunctional mRNA maturation, which is observed for pseudo-operon configurations in yeast [27].

The different mutants strongly varied in DHA accumulation (Figs. 3, 4). The configuration with blocks of four UAS1B elements upstream of the *P*_*minLEU2*_ promoter, an intergenic spacer upstream of the promoter, and the intron worked best. The corresponding mutant accumulated 20% more DHA than the second-best strain and 16-fold more than the basic strain that expressed a minimal cluster. This finding demonstrated that DHA production efficiency was transcriptionally limited. Furthermore, it revealed the potential of streamlining the promoter architecture of bacterial multigene clusters for optimized performance in *Y. lipolytica*.

As shown, strains that expressed the DHA cluster under the control of *P*_*TEF*_ yielded less product (Fig. 3). This finding was surprising, given that *P*_*TEF*_ mediates strong constitutive expression and is often preferred for metabolic engineering of *Y. lipolytica* [35, 36, 38]. Here, it was indeed active in all culture phases, but *P*_*TEF*_*-*driven expression fluctuated over time (Fig. 6). A previous study of the related promoter of the gene *TEF1* in *Pichia pastoris* encoding the translation elongation factor alpha-1 revealed growth-associated expression [39]. In this context, the expression of the cluster under the control of *P*_*TEF*_ might not have optimally matched DHA biosynthesis, occurring only during the stationary phase (Fig. 4). On the other hand, the intuitive use of *P*_*minLEU2*_ in the initial DHA producer [20] proved in retrospect to be a good choice.

### The iterative use of UAS1B16 tandem repeats within the multigene cluster causes genetic instability

As shown, the use of UAS1B elements strongly enhanced the expression of all cluster genes (Fig. 5A, B), matching the previous observation that *Yarrowia* promoters are enhancer limited [36]. Functional clusters with blocks of sixteen elements in front of each cluster gene enabled the highest DHA production, as observed during the early phase of production (Additional file 1: Figures S1D, E). However, these cluster configurations were found to be unstable over time. Recombination events resulted in the loss of genes, preferentially the genes in the middle of the cluster. In rare cases, the first gene, *pfa1,* was also lost, whereas *ppt*, located outside the potentially recombining homology domains, was stable. As shown, only a subpopulation of cells could form the product during later stages of the process (Fig. 5C). The additional repetitive use of the *TEF* intron destabilized the cluster further (Fig. 5). Strain *U16 TEF INT* Af4 even failed to produce DHA in the main culture. Almost all cells exhibited a damaged PUFA cluster during the early stages of the production process. At a low frequency, mutants with four tandem repeats also lost parts of the cluster (Fig. 5). Without doubt, genetic stability is a crucial factor in microbial cell factories to ensure reproducible performance and constant product quality [40], but little has been reported on genetic stability in *Y. lipolytica*. Notably, cloning of the five-step violacein pathway yielded phenotypically different *Y. lipolytica* clones [27], while environmental stress factors caused the emergence of less-producing cell subpopulations in recombinant protein-producing *Y. lipolytica* expressing single genes [41].

Here, the major reason for genetic instability was the four UAS1B16 blocks present in the cluster at a distance between 2.1 and 8.4 kb. Previously, UAS1B tandem repeats have been identified as powerful elements that enhance gene expression in *Y. lipolytica* [36]. Single UAS1B12 and UAS1B16 copies were analysed for genetic stability given their highly repetitive nature [36]. After 192 h of nonselective culturing (36 doublings), 17 out of 20 UAS1B12-based plasmids were found intact, while in 3 cases, the tandem repeats were truncated down to UAS1B3. Overall, this suggested relatively high stability of the elements themselves. In contrast, our findings show that repetitive UAS1B16 blocks in certain proximity, meant to drive individual promoters in synthetic multigene clusters, should be avoided in *Y. lipolytica*. Additional repetitive elements such as promoters, introns and spacers may decrease genetic stability further.

### PUFA gene clusters with iterative UAS1B4 tandem repeats enable stable expression over two weeks of culture

Naturally, *Y. lipolytica* carries only a single copy of UAS1B as a proximal enhancer, together with UAS2B as a distal enhancer, to drive the expression of the *xpr*2 gene [42]. In this regard, the UAS1B16 blocks introduced in proximity were in a rather unnatural layout, which might explain the degeneration over time. Beneficially, UAS1B4 tandem repeats worked quite well and enabled a significant upregulation of expression together with stable production for up to two weeks, and these motifs can be recommended. In any case, genetic stability monitoring of genome-based *Y. lipolytica* strains cultured under nonselective conditions seems important when evaluating recombinant producers to avoid errors in the interpretation of metabolic engineering efforts [43]. Notably, the degenerated strains did not display any visible phenotypic differences (except lower DHA titres). The discovery of genetic instability was therefore essential to understand why the apparently “strongest” clusters performed weakly.

### The 5’ TEF intron reveals gene-specific positional effects enabling the rebalancing of gene expression within the PUFA cluster

Efficient refactoring of biosynthesis-related gene clusters (BGCs) in heterologous hosts can be important to achieve high production efficiency [44], as supported by a range of prominent examples [32, 45–50]. Approximately 15% of the genes of *Y. lipolytica* contain introns [51], rendering this element important for expression control in yeast [25]. Here, the introduction of the *TEF* intron resulted in a mixed outcome (Fig. 6C, D, Additional file 1: Figure S2). Similarly, previous studies revealed a complex picture. For example, the *TEF* intron had a positive influence on GFP expression for multiple promoters, including *P*_*TEF,*_ but caused no effect when using the *GPD* promoter [52]. In another study designed to increase fatty ester production, the *TEF* intron revealed strongly reduced gene expression under the control of UAS1B4 *P*_*TEF*_, whereas the combination with the core *P*_*TEF*_ promoter had no influence [53]. Moreover, introns revealed positional effects when integrated in two-gene configurations [27]. Here, we observed significant promoter- and gene-specific effects of the *TEF* intron (Fig. 6C, D, Additional file 1: Figure. S3B, C and D). It modulated the expression of the cluster genes when combined with the *TEF* promoter but caused only marginal effects in* P*_*minLEU2*_-based mutants (Fig. 6, Additional file 1: Figure S3B, C and D). Regarding the cluster genes, the TEF intron reduced the expression of the first and last genes, *pfa1* and *ppt*, while amplifying the expression of the two middle genes, *pfa2* and *pfa3,* in different phases of cultivation (Fig. 6). Clearly, the functionality of the intron depended on the genetic environment within the PUFA cluster, differing from the UAS1B elements, which enabled generally increased gene expression. Both elements were found to yield synergistic effects (Fig. 3). As shown, a strong total expression of the PUFA cluster genes (Fig. 7D) as well as a high expression ratio of (*pfa2* + *pfa3*) versus (*pfa1* + *ppt*) (Fig. 7E) emerged as important features of high-level producers. In addition, we found positive effects on DHA levels when introducing a spacer between genes (Fig. 3). Interestingly, the positional effect of individual genetic elements was also observed during metabolic engineering of *Y. lipolytica* to produce extracellular hydrolases, underlining the impact of the architecture of complex expression cassettes on strain performance [54].

In addition, the findings are interesting also from the metabolic engineering perspective. Regarding lipid synthesis, up regulation of the first and the last genes of the metabolic pathway involved, was indicated as the best strategy for amplifying flux [55]. While the engineered pathway in the previous work comprised a linear cascade of individual enzymes, our study was based on an enzyme complex that catalyzed the major synthetic steps through ten iterative cycles instead. While the success of the “push-and-pull” approach at both ends can be well explained for the linear pathway, a straightforward explanation cannot be given for the cyclic PKS-based DHA synthesis, given the fact that the genes *pfa1, pfa2,* and *pfa3*, each comprised several catalytic modules and that these acted multiple times on the growing fatty acid (Fig. 1). In this regard, further efforts in rebalancing expression of the PKS-based cluster might contribute to better understand the interplay.

### The extent of DHA accumulation correlates with the availability of intracellular CoA esters

The increase in cluster expression increased DHA formation, demonstrating that the efficiency of the initially constructed strains was transcriptionally limited (Fig. 3). Additionally, sufficient precursor availability is an important prerequisite for efficient metabolite overproduction [56–59]. The synthesis of one molecule of DHA requires the supply of 1 molecule of acetyl-CoA and ten molecules of malonyl-CoA. It was therefore interesting to note that strains with higher DHA production contained significantly fewer intracellular CoA esters (Fig. 7A). The pool of malonyl-CoA, the major DHA building block, was even closely correlated with the extent of overproduction: high-level producers contained sixfold less CoA thioester (Fig. 7C). Notably, the pools of succinyl-CoA and malonyl-CoA were also found to be coupled, indicating balancing effects between different CoA esters, which was also observed in other microbes (Fig. 7A) [58, 60, 61]. The findings indicate streamlining the CoA ester supply for further strain improvement, which has proven successful before in overproducing other CoA-based products in *Y. lipolytica* [62–64].

## Conclusions

In this work, we studied docosahexaenoic acid (DHA) production in *Y. lipolytica* using a modular cloning approach to optimize the expression of a four-gene cluster encoding a PKS-like PUFA synthase. As shown, DHA synthesis was transcriptionally limited, as enhanced transcription led to enhanced formation of the product. The systematic combination of upstream activating sequence elements, promotors, introns, and spacers underlined the impact of the various elements on expression control. The stationary phase *minLEU2* promoter was found much stronger than the constitutive *TEF* promoter. Likely driven by the dynamic properties of *P*_*minLEU2*_, DHA production was highest during the stationary phase, opening further space for optimization, if the cluster expression could be extended into the growth phase [65]. The regulatory elements assembled within a single cistron may act synergistically (UAS1B plus intron in combination with *P*_*TEF*_; intron plus spacer in combination with *P*_*minLEU2*_) or render no effect (intron and *P*_*minLEU2*_) suggesting combinatorial testing, as done here, to find well performing combinations.

The expression ratio between the four genes of the synthetic cluster strongly modulated DHA production efficiency, as visualized by the strong boost in DHA production in strains with high expression levels of the two middle genes and low expression levels of the outer genes. Furthermore, the instability of large expression cassettes bearing repetitive elements is an important negative factor, affecting the performance of producing strains, and hence should be monitored carefully.

For future studies, several options appear promising to further improve the PUFA cluster architecture and enable stable high-level expression, even beyond the well-working monocistronic UAS1B4-based design developed here. The individual cluster genes could be distributed to different loci (including different chromosomes) in the genome to minimize/exclude recombination events. Such a layout should be stable, even when used with blocks of sixteen or more UAS1B tandem repeats [36]. This strategy would be linked to increased effort in finding suitable expression loci and constructing the producers stepwise but appears promising given the fast and powerful genomic engineering technologies for *Y. lipolytica* [27, 38, 66, 67]. One might also think about expressing the cluster from two sequence blocks with two convergently (tail-to-tail) oriented genes each to maximize the distance between the repetitive elements and further enhance expression [68]. In addition, strains based on UAS1B6, UAS1B8 or UAS1B10 blocks could provide a useful compromise between expression strength and stability. One could also try to express the cluster as a bacterial-type polycistronic operon using a single promoter driven by only one large UAS1B element, although this architecture seems inferior to the monocistronic design in yeast [27].

Interestingly, the stepwise increase of DHA production was linked to gradually reduced availability of acetyl-CoA and malonyl-CoA, pointing to bottlenecks in precursor supply in the best producers and suggesting a more global consideration of metabolic pathways towards further strain improvement [57, 69–72]. Regarding production, process conditions and environmental stress appear relevant to be studied further, given their impact on strain performance [41, 71, 73–75]. These studies might include the aspect of morphology, shown to play a crucial role for the metabolism of filamentous microbes [76–79], including dimorphic *Y. lipolytica* [41]. Beyond the production of DHA, our findings may provide valuable guidance to streamline other *Y. lipolytica* cell factories based on heterologous multigene pathways [62, 63, 80].

## Materials and methods

### Plasmids and strains

*Escherichia* *coli* DH10B (Thermo Fisher Scientific, Waltham, MA, USA) was used for cloning purposes. The basic *Y. lipolytica* Po1h (CLIB 882) was taken from previous work [20]. Strains were maintained as glycerol stocks at −80 °C. The plasmids pUC19 (NEB, Ipswich, MA, USA), pACYC_assembly, pKG2-PIS and pSynPfaPptAf4 [20] were used for cluster assembly. All strains and plasmids used are listed in the supplement (Additional file 1: Tables S2, S3).

### Molecular design and genetic engineering

For the design of cloning strategies, the software SnapGene 6 (Insightful Science, San Diego, CA, USA) was used. The workflow for the assembly of different PUFA cluster variants was as follows. In the first step, the gene sequence of interest and the sequence of the Lip2t terminator were amplified from the plasmid pSynPfaPptAf4. The corresponding promoter sequences were synthetized (GenScript, Piscataway Township, NJ, USA). The different elements (promoter, gene, and terminator) were then fused and integrated into the linearized vector pUC19 using restriction enzymes and Gibson assembly. Optionally, the respective number of UAS1B tandem repeats was also integrated (SmaI). PCR and sequencing were conducted to verify the correctness of each construct (Genewiz, Leipzig, Germany). The plasmids were transformed into *E. coli* DH 10B by heat shock for amplification and isolation [81].

Four single-gene constructs were then assembled and integrated into pACYC_assembly. The correct order of *pfa1, pfa2, pfa3*, and *ppt* was achieved using selective restriction (SdaI/ApaLI, AvrII/PacI, ApaLI/AclI, AclI/PacI, FastDigest Enzymes, Thermo Fisher) and ligation cycles (Fast-Link DNA Ligation Kit, Lucigen, Middleton, WI, USA). The plasmids were transformed into *E. coli* DH 10B by heat shock for amplification and isolation [81]. The obtained four-gene clusters were validated by PCR and then integrated into the plasmid pKG2-PIS using restriction (SdaI/PacI, Thermo Fisher) and ligation (Lucigen). The plasmid was amplified and isolated as described above and was then once again validated by PCR. Subsequently, the vector was linearized (SmiI/NotI, Thermo Fisher). The linearized DNA was integrated into *Yarrowia lipolytica* Po1h (YALI0_C05907g) using lithium acetate-mediated heat shock transformation [82]. In short, competent cells were prepared by resuspending 5*10^7^ cells in 600 µL of lithium acetate (LiAc) solution (0.1 M, pH 6.0), followed by incubation for 1 h at 28 °C. Afterwards, the cells were harvested (4000  ×  *g*, 1 min, RT), resuspended in 40 µL of LiAc solution, amended with 10 µL of herring testes carrier DNA (10 mg mL^−1^ in TE buffer, denatured) and 500 ng of linearized target DNA, and incubated for 15 min at 28 °C. Afterwards, 350 µL of the LiAc-PEG solution (40% PEG 4000 in 0.1 M lithium acetate, pH 6.0) was added, followed by further incubation for 1 h at 28 °C. Finally, 40 µL of DMSO was added, and the mixture was heat shocked at 39 °C for 10 min, resuspended in 600 µL LiAc solution, and plated on YNB-N_5k_. After 3–4 days of incubation at 28 °C, the grown colonies were checked for correct integration by colony PCR. A positive clone was additionally checked for the presence of each of the four PUFA genes and qualitatively evaluated for DHA production using GC‒MS analysis of its fatty acid composition after culturing in glycerol-based medium (see below) prior to cryo-conservation.

### Growth media

*Yarrowia lipolytica* Po1h was grown in complex YPD medium containing 10 g L^−1^ of yeast extract (Becton Dickinson, Heidelberg, Germany), 20 g L^−1^ of peptone (Becton Dickinson), and 22 g L^−1^ of glucose. All other *Y. lipolytica* strains were grown in a chemically defined medium (YNB-N5000) containing 1.7 g L^−1^ of YNB (Sigma-Aldrich, Darmstadt, Germany), 10 g L^−1^ glycerol, 5 g L^−1^ (NH_4_)_2_SO_4_ and 200 mM MES (pH 6.7). For solid plate cultures, 20 g L^−1^ agar (Becton Dickinson) was added. For plasmid selection, 100 µg mL^−1^ ampicillin, 25 µg mL^−1^ chloramphenicol, or 50 µ mL^−1^ kanamycin was added.

### Cultivation

Cultivation experiments were conducted in 500 mL baffled shake flasks filled with 50 mL of medium and incubated on an orbital shaker (5 cm shaking diameter, 230 rpm, Multitron, Infors AG, Bottmingen, Switzerland) at 28 °C and 80% humidity. The preculture was inoculated with a single colony from a two-day preincubated plate culture, grown overnight, harvested (4000  ×  *g*, RT, 1 min), and used to inoculate the main culture to a starting OD_600_ of 0.1. All cultivations were conducted in biological triplicate.

### Determination of the cell concentration

The cell concentration was inferred from photometric measurement at 600 nm. An experimentally obtained correlation for *Y. lipolytica* allowed us to infer the concentration of the cell dry mass (CDM) from OD_600_ readings: CDM [g L^−1^] = 0.424 × OD_600_ [83].

### Quantification of glycerol and citrate

Glycerol and citrate were quantified by HPLC (Agilent 1200 series, Agilent Technologies, Waldbronn, Germany) using an anion exchange column (300 × 7.8 mm, Aminex HPX-87H, Bio-Rad, Hercules, CA, USA) at 45 °C and 12 mM H_2_SO_4_ at a flow rate of 0.5 mL min^−1^. The analytes were detected via refractive index measurement and quantified using external standards. The method also allowed the assessment of other organic acids and alcohols. Such compounds, however, were not detected in any culture.

### Quantification of phosphate

Ion chromatography (Dionex Integrion, Thermo Scientific) was used to quantify phosphate in culture samples. The setup included a carbonate-selective anion-exchange column (IonPac AG9-HC, IonPac AS9-HC, Dionex Integrion) at 35 °C as the stationary phase and 12 mM Na_2_CO_3_ (0.25 mL min^−1^) as the mobile phase and was operated with eluent suppression (ERS 500 suppressor, 20 mA, Dionex Integrion). Phosphate was detected using conductivity analysis and quantified via external standards.

### Extraction and transesterification of fatty acids

CDM (5 mg) was transferred into a glass vial, collected (12,000  ×  *g*, 4 °C, 5 min), and dried in a vacuum concentrator (Savant DNA 120 SpeedVac, Thermo Fisher) for 60 min at 65 °C and 9 mbar. Then, 300 µL of a mixture of methanol, toluene, and 95% sulfuric acid (50:50:2; v/v/v) was added for simultaneous extraction and transesterification into the corresponding fatty acid methyl esters (FAMEs). Moreover, *n*-3 heneicosapentaenoic acid methyl ester (HPA, 22: 5, Cayman Chemical, Ann Arbor, MI, USA) was added as an internal standard. The mixture was incubated at 80 °C for 24 h. After cooling to room temperature, the reaction was neutralized with 250 µL of a stopping solution (0.5 M NH_4_HCO_3_ and 2 M KCl in H_2_O). After phase separation (12,000×*g*, RT, 5 min), the organic phase was taken for further analysis.

### Analysis of FAMEs by GC‒MS

The analysis was conducted on a GC‒MS instrument (6890 GC, 5973 inert MSD, Agilent Technologies), with which the FAMEs were separated on a fused silica high-polarity column (HP-88, 30 m, 0.25 mm, 0.2 µm, Agilent Technologies). Samples (1 µL) were injected at a 5: 1 split ratio using helium as the carrier gas. The column was initially kept at 110 °C for 1 min and then heated to 240 °C at a rate of 4 °C min^−1^. The injector, MS transfer line, ion source, and quadrupole were kept at 250 °C, 280 °C, 230 °C, and 150 °C, respectively. After a 5 min solvent delay, the mass detector was operated in scan mode (*m/z* 25–500). The analytes were identified based on retention time and fragmentation pattern using a mixed synthetic standard (Supelco 37 Component FAME Mix, Sigma-Aldrich) and pure DHA (Cayman Chemical). Quantification was based on HPA as an internal standard.

### Extraction and quantification of intracellular CoA thioesters

The analysis of CoA thioesters was performed as described previously [65]. In short, broth (containing 8 mg of CDM) was transferred into four volumes of an extraction and quenching buffer (95% acetonitrile, 25 mM formic acid, −20 °C), followed by the addition of an internal ^13^C-enriched CoA thioester standard. After incubation for 10 min on ice, the solution was centrifuged to remove debris (15,000×*g*, 4 °C, 10 min). The obtained supernatant was mixed with 10 mL of ice-cold deionized water. The residual pellet was washed twice with 8 mL of ice-cold deionized water. All extracts were combined, frozen in liquid nitrogen, and freeze-dried. The obtained pellet was dissolved in 500 µL of buffer (25 mM ammonium formate, pH 3.0, 2% methanol, 4 °C), and the obtained solution was filtered (Ultrafree-MC, GV 0.22 µm, Millipore, Germany). The CoA thioesters in the filtrate were analysed by LC‒ESI‒MS/MS (Agilent Infinity 1290 System, QTRAP 6500 + , AB Sciex, Darmstadt, Germany) [60]. Here, separation was based on a core–shell reversed-phase column (Kinetex XB-C18, 100 × 2.1 mm, 2.6 μm, 100 Å, Phenomenex) at 40 °C as the stationary phase and a gradient of formic acid (50 mM, adjusted to pH 8.1 with 25% ammonium hydroxide, eluent A) and methanol (eluent B) as the mobile phase at 300 µL min^−1^: 0–7 min, 0–10% B; 7–10 min, 10–100% B; 10–11 min, 100% B; 11–12 min, 100–0% B; 12–15 min, 0% B. Multiple reaction monitoring (MRM) was used for detection. Samples were analysed in biological triplicate.

### Gene expression analysis using real-time PCR (qRT‒PCR)

The analysis was based on a previously developed protocol [65]. Cells were quickly collected by centrifugation (20,000×*g*, 4 °C, 1 min), and the obtained pellet was immediately frozen in liquid nitrogen. Total RNA was isolated using the RiboPure RNA Purification Kit (Invitrogen). RNA concentration and RNA quality were evaluated (NanoDrop 1000, Thermo Scientific, Agilent 2100 Bioanalyzer, RNA 6000 Nano Kit, Agilent Technologies). Then, the RNA (250 mg) was converted into cDNA (Maxima First Strand Kit, with dsDNase, Thermo Scientific). The cDNA samples were diluted 1:100 in DEPC-treated water (Invitrogen, Waltham, MA, USA). The analysis was conducted in a real-time PCR system (QuantStudio 3 System, Thermo Scientific) using PowerUp SYBR Green Master Mix (Applied Biosystems, Waltham, MA, USA) and Microamp Fast Optical 96-Well plates (Applied Biosystems). Primers were designed using Primer3Plus, Primer-BLAST, and OligoCalc and experimentally validated by PCR and a standard curve test [65]. For normalization, the 25S rRNA gene (YalifMr30) was used [65]. Samples were analysed in biological triplicate.

### Supplementary Information

**Additional file 1: Figure S1.** Impact of the genetic architecture of heterologous PUFA clusters on the production of docosahexaenoic acid **(**DHA) in recombinant *Y. lipolytica*. The strains were cultivated in glycerol-based minimal medium. The time point of glycerol depletion is indicated by a dotted line. The different genetic cluster layouts can be taken from Fig. 3. NFA = native fatty acids. n = 3. **Figure S2.** Relative expression strength of the PUFA cluster genes, normalized to the expression level of *pfa1* of strain *TEF* Af4, which was set to one. The expression values display the average from three sampling time points at x, y, and z h. n = 3. **Figure S3.** Influence of genetic elements on the expression level of the PUFA cluster genes during the exponential phase (ca. 15 h), early stationary phase (ca. 38 h), and late stationary phase (ca. 136 h). The data show the comparison between strains *TEF INT* Af4 (left bars) and *U4 TEF INT* Af4 (right bars) (**A**). The values were normalized to the expression level of each gene in *TEF* Af4, which was set to one. In addition, the data show the relative expression levels of the individual genes in the strains *U4 TEF* Af4 (left bars) and *U4 TEF INT* Af4 (right bars) over time (**B**). Here, the relative expression of a single gene is given against the total PUFA gene expression for each time point. Furthermore, expression data are given for the strains U4 minLEU2 Af4 (left bars) and *U4 minLEU2 INT* Af4 (right bars) (**C**), as well as *U4 minLEU2 S* Af4 (left bars) and *U4 minLEU2 INT S* Af4 (right bars) (**D**). Statistical significance was calculated with Student’s t test. *: p = 0.05; **: p = 0,01. n = 3. **Table S1.** Growth and DHA production performance in different glycerol-grown strains of *Y. lipolytica*. The data show the final values after 185 h. n = 3. **Table S2.** Strains used in this study. **Table S3.** Plasmids used in this study. **Table S4.** Assembly and sequencing primers. Overlaps are shown in bold, and restriction sites are underlined. **Table S5.** Generated genetic building blocks for cluster assembly. Table S6: Generated PUFA clusters.

## Data Availability

The dataset(s) supporting the conclusions of this article are all included within the article.

## References

[1] Shahidi F, Ambigaipalan P (2018). Omega-3 polyunsaturated fatty acids and their health benefits. Annu Rev Food Sci Technol.

[2] Li J, Pora BLR, Dong K, Hasjim J (2021). Health benefits of docosahexaenoic acid and its bioavailability: a review. Food Sci Nutr.

[3] Chiu C-C, Su K-P, Cheng T-C, Liu H-C, Chang C-J, Dewey ME, Stewart R, Huang S-Y (2008). The effects of omega-3 fatty acids monotherapy in Alzheimer's disease and mild cognitive impairment: a preliminary randomized double-blind placebo-controlled study. Prog Neuropsychopharmacol Biol Psychiatry.

[4] Hussein N, Ah-Sing E, Wilkinson P, Leach C, Griffin BA, Millward DJ (2005). Long-chain conversion of [13C]linoleic acid and α-linolenic acid in response to marked changes in their dietary intake in men. J Lipid Res.

[5] Gladyshev MI, Sushchik NN, Makhutova ON (2013). Production of EPA and DHA in aquatic ecosystems and their transfer to the land. Prostaglandins Other Lipid Mediat.

[6] Colombo SM, Rodgers TFM, Diamond ML, Bazinet RP, Arts MT (2020). Projected declines in global DHA availability for human consumption as a result of global warming. Ambio.

[7] Jovanovic S, Dietrich D, Becker J, Kohlstedt M, Wittmann C (2021). Microbial production of polyunsaturated fatty acids—high-value ingredients for aquafeed, superfoods, and pharmaceuticals. Curr Opin Biotechnol.

[8] Georgiadis I, Tsiligkaki C, Patavou V, Orfanidou M, Tsoureki A, Andreadelli A, Theodosiou E, Makris AM (2023). Identification and construction of strong promoters in *Yarrowia*
*lipolytica* suitable for glycerol-based bioprocesses. Microorganisms.

[9] Beopoulos A, Cescut J, Haddouche R, Uribelarrea J-L, Molina-Jouve C, Nicaud J-M (2009). *Yarrowia lipolytica* as a model for bio-oil production. Prog Lipid Res.

[10] Ledesma-Amaro R, Nicaud J-M (2016). *Yarrowia lipolytica* as a biotechnological chassis to produce usual and unusual fatty acids. Prog Lipid Res.

[11] Nicaud JM (2012). *Yarrowia*
*lipolytica*. Yeast.

[12] Groenewald M, Boekhout T, Neuvéglise C, Gaillardin C, van Dijck PW, Wyss M (2014). *Yarrowia lipolytica*: safety assessment of an oleaginous yeast with a great industrial potential. Crit Rev Microbiol.

[13] Jost B, Holz M, Aurich A, Barth G, Bley T, Muller RA (2015). The influence of oxygen limitation for the production of succinic acid with recombinant strains of *Yarrowia*
*lipolytica*. Appl Microbiol Biotechnol.

[14] Ledesma-Amaro R, Dulermo T, Nicaud JM (2015). Engineering *Yarrowia*
*lipolytica* to produce biodiesel from raw starch. Biotechnol Biofuels.

[15] Darvishi F, Nahvi I, Zarkesh-Esfahani H, Momenbeik F (2009). Effect of plant oils upon lipase and citric acid production in *Yarrowia*
*lipolytica* yeast. J Biomed Biotechnol.

[16] Darvishi F, Ariana M, Marella ER, Borodina I (2018). Advances in synthetic biology of oleaginous yeast *Yarrowia*
*lipolytica* for producing non-native chemicals. Appl Microbiol Biotechnol.

[17] Cordova LT, Alper HS (2018). Production of α-linolenic acid in *Yarrowia lipolytica* using low-temperature fermentation. Appl Microbiol Biotechnol.

[18] Sun M-L, Madzak C, Liu H-H, Song P, Ren L-J, Huang H, Ji X-J (2017). Engineering *Yarrowia lipolytica* for efficient γ-linolenic acid production. Biochem Eng J.

[19] Xue Z, Sharpe PL, Hong S-P, Yadav NS, Xie D, Short DR, Damude HG, Rupert RA, Seip JE, Wang J (2013). Production of omega-3 eicosapentaenoic acid by metabolic engineering of *Yarrowia lipolytica*. Nat Biotechnol.

[20] Gemperlein K, Dietrich D, Kohlstedt M, Zipf G, Bernauer HS, Wittmann C, Wenzel SC, Müller R (2019). Polyunsaturated fatty acid production by* Yarrowia lipolytica* employing designed myxobacterial PUFA synthases. Nat Commun.

[21] Gemperlein K, Rachid S, Garcia RO, Wenzel SC, Müller R (2014). Polyunsaturated fatty acid biosynthesis in myxobacteria: different PUFA synthases and their product diversity. Chem Sci.

[22] Schwartz C, Shabbir-Hussain M, Frogue K, Blenner M, Wheeldon I (2017). Standardized markerless gene integration for pathway engineering in *Yarrowia lipolytica*. ACS Synth Biol.

[23] Holkenbrink C, Dam MI, Kildegaard KR, Beder J, Dahlin J, Doménech Belda D, Borodina I (2018). EasyCloneYALI: CRISPR/Cas9-based synthetic toolbox for engineering of the yeast *Yarrowia lipolytica*. Biotechnol J.

[24] Shabbir Hussain M, Gambill L, Smith S, Blenner MA (2016). Engineering Promoter Architecture in Oleaginous yeast *Yarrowia*
*lipolytica*. ACS Synth Biol.

[25] Le Hir H, Nott A, Moore MJ (2003). How introns influence and enhance eukaryotic gene expression. Trends Biochem Sci.

[26] Portela RM, Vogl T, Kniely C, Fischer JE, Oliveira R, Glieder A (2017). Synthetic core promoters as universal parts for fine-tuning expression in different yeast species. ACS Synth Biol.

[27] Wong L, Engel J, Jin E, Holdridge B, Xu P (2017). YaliBricks, a versatile genetic toolkit for streamlined and rapid pathway engineering in *Yarrowia lipolytica*. Metab Eng Commun.

[28] Nicaud J-M, Madzak C, van den Broek P, Gysler C, Duboc P, Niederberger P, Gaillardin C (2002). Protein expression and secretion in the yeast Yarrowia lipolytica. FEMS Yeast Res.

[29] Song W, Li J, Liang Q, Marchisio MA (2016). Can terminators be used as insulators into yeast synthetic gene circuits?. J Biol Eng.

[30] Schwechheimer SK, Becker J, Peyriga L, Portais JC, Sauer D, Muller R, Hoff B, Haefner S, Schroder H, Zelder O, Wittmann C (2018). Improved riboflavin production with *Ashbya gossypii* from vegetable oil based on (13)C metabolic network analysis with combined labeling analysis by GC/MS, LC/MS, 1D, and 2D NMR. Metab Eng.

[31] Hoffmann SL, Kohlstedt M, Jungmann L, Hutter M, Poblete-Castro I, Becker J, Wittmann C (2021). Cascaded valorization of brown seaweed to produce l-lysine and value-added products using *Corynebacterium glutamicum* streamlined by systems metabolic engineering. Metab Eng.

[32] Rohles C, Pauli S, Giesselmann G, Kohlstedt M, Becker J, Wittmann C (2022). Systems metabolic engineering of Corynebacterium glutamicum eliminates all by-products for selective and high-yield production of the platform chemical 5-aminovalerate. Metab Eng.

[33] Weiland F, Barton N, Kohlstedt M, Becker J, Wittmann C (2023). Systems metabolic engineering upgrades Corynebacterium glutamicum to high-efficiency cis, cis-muconic acid production from lignin-based aromatics. Metab Eng.

[34] Jovanovic S, Dietrich D, Becker J, Kohlstedt M, Wittmann C (2021). Microbial production of polyunsaturated fatty acids - high-value ingredients for aquafeed, superfoods, and pharmaceuticals. Curr Opin Biotechnol.

[35] Dulermo R, Brunel F, Dulermo T, Ledesma-Amaro R, Vion J, Trassaert M, Thomas S, Nicaud J-M, Leplat C (2017). Using a vector pool containing variable-strength promoters to optimize protein production in *Yarrowia*
*lipolytica*. Microb Cell Fact.

[36] Blazeck J, Liu L, Redden H, Alper H (2011). Tuning gene expression in *Yarrowia lipolytica* by a hybrid promoter approach. Appl Environ Microbiol.

[37] Shearwin KE, Callen BP, Egan JB (2005). Transcriptional interference—a crash course. Trends Genet.

[38] Sun ML, Shi TQ, Lin L, Ledesma-Amaro R, Ji XJ (2022). Advancing *Yarrowia*
*lipolytica* as a superior biomanufacturing platform by tuning gene expression using promoter engineering. Bioresour Technol.

[39] Ahn J, Hong J, Lee H, Park M, Lee E, Kim C, Choi E, Jung J, Lee H (2007). Translation elongation factor 1-alpha gene from pichia pastoris: molecular cloning, sequence, and use of its promoter. Appl Microbiol Biotechnol.

[40] Moore JC, Ramos I, Van Dien S (2022). Practical genetic control strategies for industrial bioprocesses. J Ind Microbiol Biotechnol.

[41] Gorczyca M, Kaźmierczak J, Fickers P, Celińska E (2022). Synthesis of secretory proteins in Yarrowia lipolytica: effect of combined stress factors and metabolic load. Int J Mol Sci.

[42] Blanchin-Roland S, Cordero Otero RR, Gaillardin C (1994). Two upstream activation sequences control the expression of the XPR2 gene in the yeast *Yarrowia*
*lipolytica*. Mol Cell Biol.

[43] Jones PR (2014). Genetic instability in cyanobacteria—an elephant in the room?. Front Bioeng Biotechnol.

[44] Li L, Maclntyre LW, Brady SF (2021). Refactoring biosynthetic gene clusters for heterologous production of microbial natural products. Curr Opin Biotechnol.

[45] Gießelmann G, Dietrich D, Jungmann L, Kohlstedt M, Jeon EJ, Yim SS, Sommer F, Zimmer D, Mühlhaus T, Schroda M (2019). Metabolic engineering of corynebacterium glutamicum for high-level ectoine production: design, combinatorial assembly, and implementation of a transcriptionally balanced heterologous ectoine pathway. Biotechnol J.

[46] Birchler JA, Veitia RA (2010). The gene balance hypothesis: implications for gene regulation, quantitative traits and evolution. New Phytol.

[47] Shao Z, Rao G, Li C, Abil Z, Luo Y, Zhao H (2013). Refactoring the silent spectinabilin gene cluster using a plug-and-play scaffold. ACS Synth Biol.

[48] Montiel D, Kang HS, Chang FY, Charlop-Powers Z, Brady SF (2015). Yeast homologous recombination-based promoter engineering for the activation of silent natural product biosynthetic gene clusters. Proc Natl Acad Sci U S A.

[49] Rohles CM, Gläser L, Kohlstedt M, Gießelmann G, Pearson S, del Campo A, Becker J, Wittmann C (2018). A bio-based route to the carbon-5 chemical glutaric acid and to bionylon-6,5 using metabolically engineered Corynebacterium glutamicum. Green Chem.

[50] Pauli S, Kohlstedt M, Lamber J, Weiland F, Becker J, Wittmann C (2023). Systems metabolic engineering upgrades *Corynebacterium*
*glutamicum* for selective high-level production of the chiral drug precursor and cell-protective extremolyte L-pipecolic acid. Metab Eng.

[51] Mekouar M, Blanc-Lenfle I, Ozanne C, Silva C, Cruaud C, Wincker P, Gaillardin C, Neuvéglise C (2010). Detection and analysis of alternative splicing in *Yarrowia*
*lipolytica* reveal structural constraints facilitating nonsense-mediated decay of intron-retaining transcripts. Genome Biol.

[52] Cui Z, Zheng H, Zhang J, Jiang Z, Zhu Z, Liu X, Qi Q, Hou J (2021). A CRISPR/Cas9-mediated, homology-independent tool developed for targeted genome integration in *Yarrowia*
*lipolytica*. Appl Environ Microbiol.

[53] Gao Q, Cao X, Huang Y-Y, Yang J-L, Chen J, Wei L-J, Hua Q (2018). Overproduction of fatty acid ethyl esters by the oleaginous yeast *Yarrowia*
*lipolytica* through metabolic engineering and process optimization. ACS Synth Biol.

[54] Celińska E, Borkowska M, Korpys-Woźniak P, Kubiak M, Nicaud J-M, Kubiak P, Gorczyca M, Białas W (2020). Optimization of *Yarrowia*
*lipolytica*-based consolidated biocatalyst through synthetic biology approach: transcription units and signal peptides shuffling. Appl Microbiol Biotechnol.

[55] Tai M, Stephanopoulos G (2013). Engineering the push and pull of lipid biosynthesis in oleaginous yeast *Yarrowia*
*lipolytica* for biofuel production. Metab Eng.

[56] Wittmann C, Weber J, Betiku E, Krömer J, Bohm D, Rinas U (2007). Response of fluxome and metabolome to temperature-induced recombinant protein synthesis in *Escherichia coli*. J Biotechnol.

[57] Kind S, Neubauer S, Becker J, Yamamoto M, Völkert M, Abendroth GV, Zelder O, Wittmann C (2014). From zero to hero—production of bio-based nylon from renewable resources using engineered *Corynebacterium glutamicum*. Metab Eng.

[58] Gläser L, Kuhl M, Stegmuller J, Ruckert C, Myronovskyi M, Kalinowski J, Luzhetskyy A, Wittmann C (2021). Superior production of heavy pamamycin derivatives using a bkdR deletion mutant of *Streptomyces*
*albus* J1074/R2. Microb Cell Fact.

[59] Christmann J, Cao P, Becker J, Desiderato CK, Goldbeck O, Riedel CU, Kohlstedt M, Wittmann C (2023). High-efficiency production of the antimicrobial peptide pediocin PA-1 in metabolically engineered Corynebacterium glutamicum using a microaerobic process at acidic pH and elevated levels of bivalent calcium ions. Microb Cell Fact.

[60] Gläser L, Kuhl M, Jovanovic S, Fritz M, Vogeli B, Erb TJ, Becker J, Wittmann C (2020). A common approach for absolute quantification of short chain CoA thioesters in prokaryotic and eukaryotic microbes. Microb Cell Fact.

[61] Kuhl M, Glaser L, Rebets Y, Ruckert C, Sarkar N, Hartsch T, Kalinowski J, Luzhetskyy A, Wittmann C (2020). Microparticles globally reprogram *Streptomyces albus* toward accelerated morphogenesis, streamlined carbon core metabolism, and enhanced production of the antituberculosis polyketide pamamycin. Biotechnol Bioeng.

[62] Huang Y-Y, Jian X-X, Lv Y-B, Nian K-Q, Gao Q, Chen J, Wei L-J, Hua Q (2018). Enhanced squalene biosynthesis in *Yarrowia*
*lipolytica* based on metabolically engineered acetyl-CoA metabolism. J Biotechnol.

[63] Lu Y, Yang Q, Lin Z, Yang X (2020). A modular pathway engineering strategy for the high-level production of β-ionone in *Yarrowia*
*lipolytica*. Microb Cell Fact.

[64] Arnesen JA, Borodina I (2022). Engineering of *Yarrowia*
*lipolytica* for terpenoid production. Metab Eng Commun.

[65] Jovanovic Gasovic S, Dietrich D, Gläser L, Cao P, Kohlstedt M, Wittmann C (2023). Multi-omics view of recombinant *Yarrowia lipolytica*: enhanced ketogenic amino acid catabolism increases polyketide-synthase-driven docosahexaenoic production to high selectivity at the gram scale. Metab Eng.

[66] Markham KA, Alper HS (2018). Synthetic biology expands the industrial potential of *Yarrowia*
*lipolytica*. Trends Biotechnol.

[67] Tsirigka A, Theodosiou E, Patsios SI, Tsoureki A, Andreadelli A, Papa E, Aggeli A, Karabelas AJ, Makris AM (2023). Novel evolved *Yarrowia*
*lipolytica* strains for enhanced growth and lipid content under high concentrations of crude glycerol. Microb Cell Fact.

[68] Yeung E, Dy AJ, Martin KB, Ng AH, Del Vecchio D, Beck JL, Collins JJ, Murray RM (2017). biophysical constraints arising from compositional context in synthetic gene networks. Cell Syst.

[69] Becker J, Zelder O, Hafner S, Schroder H, Wittmann C (2011). From zero to hero-Design-based systems metabolic engineering of *Corynebacterium glutamicum* for L-lysine production. Metab Eng.

[70] Rodrigues AL, Trachtmann N, Becker J, Lohanatha AF, Blotenberg J, Bolten CJ, Korneli C, de Souza Lima AO, Porto LM, Sprenger GA, Wittmann C (2013). Systems metabolic engineering of *Escherichia coli* for production of the antitumor drugs violacein and deoxyviolacein. Metab Eng.

[71] Beckers V, Poblete-Castro I, Tomasch J, Wittmann C (2016). Integrated analysis of gene expression and metabolic fluxes in PHA-producing *Pseudomonas putida* grown on glycerol. Microb Cell Fact.

[72] Rohles CM, Giesselmann G, Kohlstedt M, Wittmann C, Becker J (2016). Systems metabolic engineering of *Corynebacterium glutamicum* for the production of the carbon-5 platform chemicals 5-aminovalerate and glutarate. Microb Cell Fact.

[73] Kohlstedt M, Sappa PK, Meyer H, Maass S, Zaprasis A, Hoffmann T, Becker J, Steil L, Hecker M, van Dijl JM (2014). Adaptation of *Bacillus subtilis* carbon core metabolism to simultaneous nutrient limitation and osmotic challenge: a multi-omics perspective. Environ Microbiol.

[74] Schwechheimer SK, Becker J, Peyriga L, Portais JC, Sauer D, Müller R, Hoff B, Haefner S, Schröder H, Zelder O, Wittmann C (2018). Improved riboflavin production with *Ashbya gossypii* from vegetable oil based on ^13^C metabolic network analysis with combined labeling analysis by GC/MS, LC/MS, 1D, and 2D NMR. Metab Eng.

[75] Kohlstedt M, Weimer A, Weiland F, Stolzenberger J, Selzer M, Sanz M, Kramps L, Wittmann C (2022). Biobased PET from lignin using an engineered cis, cis-muconate-producing *Pseudomonas*
*putida* strain with superior robustness, energy and redox properties. Metab Eng.

[76] Driouch H, Sommer B, Wittmann C (2010). Morphology engineering of *Aspergillus niger* for improved enzyme production. Biotechnol Bioeng.

[77] Driouch H, Hansch R, Wucherpfennig T, Krull R, Wittmann C (2012). Improved enzyme production by bio-pellets of* Aspergillus niger*: targeted morphology engineering using titanate microparticles. Biotechnol Bioeng.

[78] Gao Q, Liu J, Liu L (2014). Relationship between morphology and itaconic acid production by *Aspergillus terreus*. J Microbiol Biotechnol.

[79] Kuhl M, Ruckert C, Glaser L, Beganovic S, Luzhetskyy A, Kalinowski J, Wittmann C (2021). Microparticles enhance the formation of seven major classes of natural products in native and metabolically engineered actinobacteria through accelerated morphological development. Biotechnol Bioeng.

[80] Arnesen JA, Kildegaard KR, Cernuda Pastor M, Jayachandran S, Kristensen M, Borodina I (2020). Yarrowia lipolytica strains engineered for the production of terpenoids. Front Bioeng Biotechnol.

[81] Inoue H, Nojima H, Okayama H (1990). High efficiency transformation of *Escherichia*
*coli* with plasmids. Gene.

[82] Barth G, Gaillardin C, Wolf K (1996). *Yarrowia**lipolytica*. Nonconventional yeasts in biotechnology: a handbook.

[83] Gläser L, Kuhl M, Jovanovic S, Fritz M, Vögeli B, Erb TJ, Becker J, Wittmann C (2020). A common approach for absolute quantification of short chain CoA thioesters in prokaryotic and eukaryotic microbes. Microb Cell Fact.

