# Supplementary material for: Refactoring the architecture of a polyketide gene cluster enhances docosahexaenoic acid production in Yarrowia lipolytica through improved expression and genetic stability
Source: Microb Cell Fact. 2023 Sep 29;22:199. doi: 10.1186/s12934-023-02209-9 (PMC10540379; doi:10.1186/s12934-023-02209-9)
Supplement: Supplementary file 1 — Additional file 1: Figure S1. Impact of the genetic architecture of heterologous PUFA clusters on the production of docosahexaenoic acid (DHA) in recombinant Y. lipolytica. The strains were cultivated in glycerol-based minimal medium. The time point of glycerol depletion is indicated by a dotted line. The different genetic cluster layouts can be taken from Fig. 3. NFA = native fatty acids. n = 3. Figure S2. Relative expression strength of the PUFA cluster genes, normalized to the expression level of pfa1 of strain TEF Af4, which was set to one. The expression values display the average from three sampling time points at x, y, and z h. n = 3. Figure S3. Influence of genetic elements on the expression level of the PUFA cluster genes during the exponential phase (ca. 15 h), early stationary phase (ca. 38 h), and late stationary phase (ca. 136 h). The data show the comparison between strains TEF INT Af4 (left bars) and U4 TEF INT Af4 (right bars) (A). The values were normalized to the expression level of each gene in TEF Af4, which was set to one. In addition, the data show the relative expression levels of the individual genes in the strains U4 TEF Af4 (left bars) and U4 TEF INT Af4 (right bars) over time (B). Here, the relative expression of a single gene is given against the total PUFA gene expression for each time point. Furthermore, expression data are given for the strains U4 minLEU2 Af4 (left bars) and U4 minLEU2 INT Af4 (right bars) (C), as well as U4 minLEU2 S Af4 (left bars) and U4 minLEU2 INT S Af4 (right bars) (D). Statistical significance was calculated with Student’s t test. *: p = 0.05; **: p = 0,01. n = 3. Table S1. Growth and DHA production performance in different glycerol-grown strains of Y. lipolytica. The data show the final values after 185 h. n = 3. Table S2. Strains used in this study. Table S3. Plasmids used in this study. Table S4. Assembly and sequencing primers. Overlaps are shown in bold, and restriction sites are underlined. Table S5. Gener [file 12934_2023_2209_MOESM1_ESM.docx]

**Additional file 1 to**

**Refactoring the architecture of a polyketide gene cluster enhances docosahexaenoic acid production in Yarrowia lipolytica through improved expression and genetic stability**

Demian Dietrich, Sofija Jovanovic Gasovic, Peng Cao, Michael Kohlstedt, and Christoph Wittmann^#^

Institute of Systems Biotechnology, Saarland University, Saarbrücken, Germany

Contact information

[demian.dietrich@uni-saarland.de](mailto:demian.dietrich@uni-saarland.de)

[sofija.jovanovic@uni-saarland.de](mailto:sofija.jovanovic@uni-saarland.de)

peng.cao@uni-saarland.de

[michael.kohlstedt@uni-saarland.de](mailto:michael.kohlstedt@uni-saarland.de)

[christoph.wittmann@uni-saarland.de](mailto:christoph.wittmann@uni-saarland.de)

^#^ Phone/FAX: +49 681 302 71970/71972

**Supplementary information on the cloning procedures**

Generation of PUFA Gene Cluster Building Blocks (BB; Table S5). The building blocks for the PUFA gene cluster were generated as follows (see Table S5 for details).

Generation of UAS and Spacer Sequences. The UAS1B4/16 sequence was excised from the plasmids pUC57-Kan-UAT4 and pUC57-Kan-UAS1B16 using BseRI, resulting in the creation of BB_U4 and BB_U16, respectively. Spacer sequences (Spacer1/2/3) were amplified from pSynPfaPptAf4 using the primer pairs Pr_Link1_FW_v2/Pr_Link1_RW_v2, Pr_Link2_FW_v2/Pr_Link2_RW_v2, and Pr_Link3_FW_v2/Pr_Link3_RW_v2 to generate BB_S1, BB_S2, and BB_S3, respectively.

Generation of Single Gene Expression Units (SGUs). Each single gene expression unit (SGU) comprises a promoter, open reading frame (ORF), and terminator sequence. PUFA genes were amplified from pSynPfaPptAf4 using PCR.

The PCR product of PUFA genes, amplified with primers Pr_Pfa1_FW_pTEF and Pr_Lip2t_RW_ApaLI_pTEFgibson, was integrated into pUC19_SdaI_PrTEF_AjuI after AjuI digestion. Subsequent digestion with SdaI+ApaLI resulted in the creation of BB_T1, and integration with SmaI led to the formation of BB_U4T1 and BB_U16T1.

Similarly, the PCR product of PUFA genes amplified with primers Pr_Pfa2_FW_pTEF and Pr_Lip2t_RW_AclI_pTEFgibson was integrated into pUC19_ApaLI_PrTEF_AjuI after AjuI digestion. Subsequent digestion with ApaLI+AclI resulted in the creation of BB_T2, and integration with SmaI led to the formation of BB_U4T2 and BB_U16T2.

The PCR product of PUFA genes amplified with primers Pr_Pfa3_FW_pTEF and Pr_Lip2t_RW_AvrII_pTEFgibson was integrated into pUC19_AclI_PrTEF_AjuI after AjuI digestion. Subsequent digestion with AclI+AvrII resulted in the creation of BB_T3, and integration with SmaI led to the formation of BB_U4T3 and BB_U16T3.

The PCR product of PUFA genes amplified with primers Pr_Ppt_FW_pTEF and Pr_Lip2t_RW_PacI_pTEFgibson was integrated into pUC19_AvrII_PrTEF_AjuI after AjuI digestion. Subsequent digestion with AvrII+PacI resulted in the creation of BB_TP, and integration with SmaI led to the formation of BB_U4TP and BB_U16TP.

Additionally, the PCR product of PUFA genes amplified with primers Pr_Pfa1_FW_pTEFin and Pr_Lip2t_RW_ApaLI was integrated into pUC19_SdaI_PrTEFin(-) after SnaBI digestion. Subsequent digestion with SdaI+ApaLI resulted in the creation of BB_Tin1, and integration with SmaI led to the formation of BB_U4Tin1 and BB_U16Tin1.

Similarly, the PCR product of PUFA genes amplified with primers Pr_Pfa2_FW_pTEFin and Pr_Lip2t_RW_AclI was integrated into pUC19_ApaLI_PrTEFin(-) after SnaBI digestion. Subsequent digestion with ApaLI+AclI resulted in the creation of BB_Tin2, and integration with SmaI led to the formation of BB_U4Tin2 and BB_U16Tin2.

The PCR product of PUFA genes amplified with primers Pr_Pfa3_FW_pTEFin and Pr_Lip2t_RW_AvrII was integrated into pUC19_AclI_PrTEFin(-) after SnaBI digestion. Subsequent digestion with AclI+AvrII resulted in the creation of BB_Tin3, and integration with SmaI led to the formation of BB_U4Tin3 and BB_U16Tin3.

Finally, the PCR product of PUFA genes amplified with primers Pr_Ppt_FW_pTEFin and Pr_Lip2t_RW_PacI was integrated into pUC19_AvrII_PrTEFin(-) after SnaBI digestion. Subsequent digestion with AvrII+PacI resulted in the creation of BB_TinP, and integration with SmaI led to the formation of BB_U4TinP and BB_U16TinP.

Additional Integrations. Furthermore, the PCR product of PUFA genes amplified with primers Pr_minLEU2_SdaI and Pr_Lip2t_RW_ApaLI_pTEF was integrated into pUC19 after SmaI digestion. Subsequent digestion with SdaI+ApaLI resulted in the formation of BB_U4M1 and BB_U16M1, respectively.

**Integration of Amplified PCR Products.** PCR products amplified using the primers Pr_minLEU2_ApaLI and Pr_Lip2t_RW_AclI_pTEFgibson were incorporated through Gibson assembly into pUC19. The resulting constructs were then cleaved using SmaI. Following this, the integration of BB_U4 or BB_U16 was performed using Gibson assembly. Subsequent digestion with ApaLI+AclI allowed for the creation of BB_U4M2 and BB_U16M2.

Similarly, the PCR product amplified with Pr_minLEU2_AclI and Pr_Lip2t_RW_AvrII_pTEFgibson was integrated via Gibson assembly into pUC19, followed by SmaI digestion. Integration of BB_U4 or BB_U16 was achieved through Gibson assembly, and further cleavage with AclI+AvrII resulted in the formation of BB_U4M3 and BB_U16M3.

Furthermore, the PCR product amplified with Pr_minLEU2_AvrII and Pr_Lip2t_RW_PacI_pTEFgibson was integrated through Gibson assembly into pUC19, which was subsequently cut with SmaI. Integration of BB_U4 or BB_U16 was performed using Gibson assembly, followed by cleavage with AvrII+PacI to create BB_U4MP and BB_U16MP.

Similarly, the PCR product amplified with Pr_Pfa1_FW_pTEFin and Pr_Lip2t_RW_ApaLI was incorporated through Gibson assembly into pUC19_SdaI_hpNdin_cassette, followed by SnaBI digestion. Subsequent SmaI digestion and Gibson assembly allowed for the integration of BB_U4 or BB_U16, followed by cleavage with SdaI+ApaLI to form BB_U4Min1 and BB_U16Min1.

Likewise, the PCR product amplified with Pr_Pfa2_FW_pTEFin and Pr_Lip2t_RW_AclI was incorporated through Gibson assembly into pUC19_ApaLI_hpNdin_cassette, followed by SnaBI digestion. Subsequent SmaI digestion and Gibson assembly facilitated the integration of BB_U4 or BB_U16, followed by cleavage with ApaLI+AclI to produce BB_U4Min2 and BB_U16Min2.

Similarly, the PCR product amplified with Pr_Pfa3_FW_pTEFin and Pr_Lip2t_RW_AvrII was incorporated through Gibson assembly into pUC19_AclI_hpNdin_cassette, followed by SnaBI digestion. Subsequent SmaI digestion and Gibson assembly allowed for the integration of BB_U4 or BB_U16, followed by cleavage with AclI+AvrII to yield BB_U4Min3 and BB_U16Min3.

Furthermore, the PCR product amplified with Pr_Ppt_FW_pTEFin and Pr_Lip2t_RW_PacI was incorporated through Gibson assembly into pUC19_AvrII_hpNdin_cassette, followed by SnaBI digestion. Subsequent SmaI digestion and Gibson assembly enabled the integration of BB_U4 or BB_U16, followed by cleavage with AvrII+PacI to create BB_U4MinP and BB_U16MinP.

**Cluster Assembly and Integration.** The PUFA gene clusters were assembled by sequentially ligating the generated building blocks into pACYC_assembly. These constructs were then cleaved using the appropriate restriction enzymes used to liberate the building blocks. Subsequently, the PUFA clusters (PC) were liberated using SdaI+PacI (refer to Table S6). The finalized PC constructs were ligated into pKG2-PIS via SdaI+PacI, resulting in the integration cassette plasmid (refer to Table S3).

For integration, the plasmids were cleaved with SwaI+NotI, and the resulting linearized integration cassette was integrated into Po1h to obtain the respective modified strains (refer to Table S2).



**
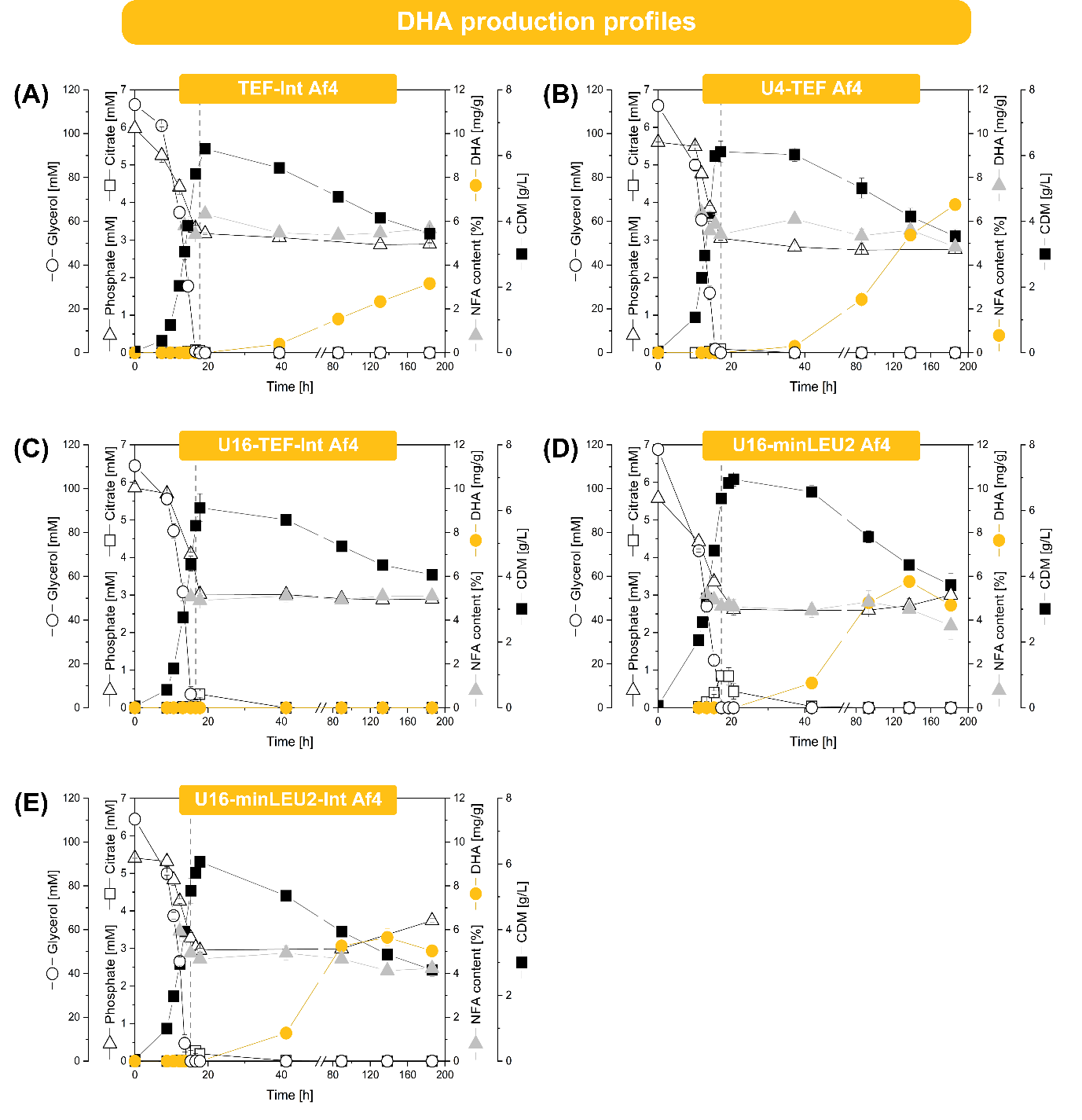
**

**Figure S1**: Impact of the genetic architecture of heterologous PUFA clusters on the production of docosahexaenoic acid (DHA) in recombinant *Y. lipolytica*. The strains were cultivated in glycerol-based minimal medium. The time point of glycerol depletion is indicated by a dotted line. The different genetic cluster layouts can be taken from Fig. 3. NFA = native fatty acids. n=3.


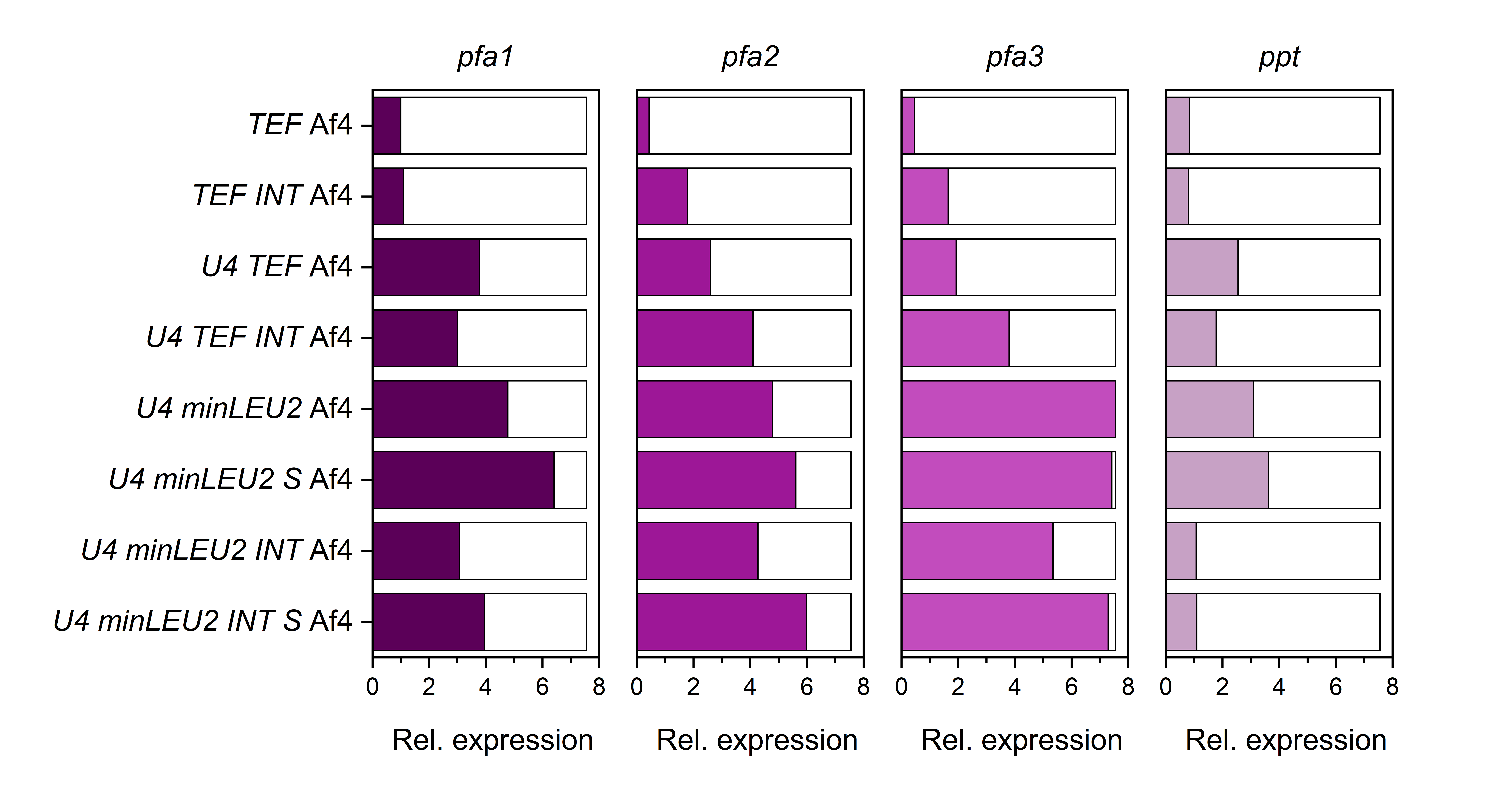


**Figure S2**: Relative expression strength of the PUFA cluster genes, normalized to the expression level of *pfa1* of the strain *TEF* Af4 which was set to one. The expression values display the average from three sampling time points at x, y, z h. n=3.





**Figure S3**: Influence of genetic elements on the expression level of the PUFA cluster genes during the exponential phase (x h), the early stationary phase (x h), and the late stationary phase (x h). The data show the comparison between strains *TEF INT* Af4 (left bars) and *U4 TEF INT* Af4 (right bars) (A). The values were normalized to the expression level of each gene in *TEF* Af4, which was set to one. In addition, the data show to the relative expression levels between the individual genes in the strains *U4 TEF* Af4 (left bars) and *U4 TEF INT* Af4 (right bars) over time (B). Hereby, the relative expression of a single gene represents is given against the total PUFA gene expression for each time point. Furthermore, expression data are given for the strains *U4 minLEU2* Af4 (left bars) and *U4 minLEU2 INT* Af4 (right bars) (C), as well as *U4 minLEU2 S* Af4 (left bars) and *U4 minLEU2 INT S* Af4 (right bars) (D). Statistical significance was calculated with a Student’s t-test. *: p=0.05; **: p=0,01. n=3.

Table S2: Strains used in this study.

| **Strains** | **Description** | **Source** |
| --- | --- | --- |
| Po1h (CLIB882) | *MatA, ura3-302, xpr2-322, axp1-2* | Madzak et al. |
| *TEF* Af4 | Po1h with a genomic copy of pTEF-*pfa1-LIP2t*-pTEF-*pfa2-LIP2t*-pTEF-*pfa3-LIP2t*-pTEF-*ppt-LIP2t (*from pPrTEFAf4) | This work |
| *U4 TEF* Af4 | Po1h with a genomic copy of U4-pTEF-*pfa1-LIP2t*-U4-pTEF-*pfa2-LIP2t*-U4-pTEF-*pfa3-LIP2t*-U4-pTEF-*ppt-LIP2t (*from pU4PrTEFAf4) | This work |
| *TEF INT* Af4 | Po1h with a genomic copy of pTEF-Int-*pfa1-LIP2t*-pTEF-Int-*pfa2-LIP2t*-pTEF-Int-*pfa3-LIP2t*-pTEF-Int-*ppt-LIP2t (*from pPrTEFinAf4) | This work |
| *U4 TEF INT* Af4 | Po1h with a genomic copy of U4-pTEF-Int-*pfa1-LIP2t*-U4-pTEF-Int-*pfa2-LIP2t*-U4-pTEF-Int-*pfa3-LIP2t*-U4-pTEF-Int-*ppt-LIP2t (*from pU4PrTEFinAf4) | This work |
| *U16 TEF INT* Af4 | Po1h with a genomic copy of U16-pTEF-Int-*pfa1-LIP2t*-U16-pTEF-Int-*pfa2-LIP2t*-U16-pTEF-INt-*pfa3-LIP2t*-U16-pTEF-Int-*ppt-LIP2t (*from pU16PrTEFinAf4) | This work |
| *U4 minLEU2* Af4 | Po1h with a genomic copy of U4-minLEU2-*pfa1-LIP2t*-U4-minLUE2-*pfa2-LIP2t*-U4-minLEU2-*pfa3-LIP2t*-U4-minLEU2-*ppt-LIP2t* (from php4dAf4) | This work |
| *U16 minLEU2* Af4 | Po1h with a genomic copy of U16-minLEU2-*pfa1-LIP2t*-U16-minLEU2-*pfa2-LIP2t*-U16-minLEU2-*pfa3-LIP2t*-U16-minLEU2-*ppt-LIP2t* (from php16dAf4) | This work |
| *U4 minLEU2 INT* Af4 | Po1h with a genomic copy of U4-minLEU2-Int-*pfa1-LIP2t*-U4-minLEU2-Int-*pfa2-LIP2t*-U4-minLEU2-Int-*pfa3-LIP2t*-U4-minLEU2-Int-*ppt-LIP2t* (from php4dinAf4) | This work |
| *U16 minLEU2 INT* Af4 | Po1h with a genomic copy of U16-minLEU2-Int-*pfa1-LIP2t*-U16-minLEU2-Int-*pfa2-LIP2t*-U16-minLEU2-Int-*pfa3-LIP2t*-U16-minLEU2-Int-*ppt-LIP2t* (from php16dinAf4) | This work |
| *U4 minLEU2 S* Af4 | Po1h with a genomic copy of U4-minLEU2-*pfa1-LIP2t-*linker1-U4-minLEU2-*pfa2*-*LIP2t*-linker2-U4-minLEU2-*pfa3*-*LIP2t*-linker3-U4-minLEU2-*ppt*-*LIP2t* (from php4dAf4_1xL) | This work |
| *U4 minLEU2 INT S* Af4 | Po1h with a genomic copy of U4-minLEU2-Int-*pfa1-LIP2t-*linker1-U4-minLEU2-Int -*pfa2*-*LIP2t*-linker2-U4-minLEU2-Int -*pfa3*-*LIP2t*-linker3-U4-minLEU2-Int -*ppt*-*LIP2t* (from php4dinAf4_1xL) | This work |

Table S3: Plasmids used in this study.

| **Plasmid** | **Description** |
| --- | --- |
| pACYC_assembly | Derivative of pACYC177 (New England Biolabs). A fragment with *SdaI, ApaLI, NcoI, SalI, AclI, AatII, NgoMIV, AvrII*, and *PacI* restriction sites were inserted into the digested plasmid (*DraI*/*BamHI*) |
| pKG2-PIS | p15A *oriV* and *cat* of pACYC184 (New England Biolabs) with  *ura3p-ura3-ura3t* and *SdaI-PacI* flanked by 1 kb homology regions for the integration in the preferred integration site. |
| pSynPfaPptAf4 | Gemperlein et al. |
| pUC57-Kan-UAT4 | Derivative of pUC57 (GenScript) with *aph(3')-Ia* kanamycin resistance gene and UAS1B2-*EcoRV*-UAS1B2 which is flanked by *BseRI* restriction sites and overlaps for the integration into *SmaI* of pUC19 by Gibson assembly. |
| pUC57-Kan-UAT4-ext | Derivative of pUC57 (GenScript) with *aph(3')-Ia* kanamycin resistance gene and UAS1B2-*EcoRV*-UAS1B2 which is flanked by *SchI* restriction sites and the integration into *EcoRV* of pUC57-Kan-UAT4 by ligation. |
| pUC57-Kan-UAS1B8 | Derivative of pUC57-Kan-UAT4 with UAS1B2-*EcoRV*-UAS1B2 from pUC57-Kan-UAT4-ext integrated into *EcoRV.* |
| pUC57-Kan-UAS1B12 | Derivative of pUC57-Kan-UAS1B8 with UAS1B2-*EcoRV*-UAS1B2 from pUC57-Kan-UAT4-ext integrated into *EcoRV.* |
| pUC57-Kan-UAS1B16 | Derivative of pUC57-Kan-UAS1B12 with UAS1B2-*EcoRV*-UAS1B2 from pUC57-Kan-UAT4-ext integrated into *EcoRV.* |
| pUC19_SdaI_PrTEF_AjuI | Derivative of pUC19 (New England Biolabs) with *SdaI-pTEF-AjuI.* |
| pUC19_ApaLI_PrTEF_AjuI | Derivative of pUC19 (New England Biolabs) with *ApaLI-pTEF-AjuI.* |
| pUC19_AclI_PrTEF_AjuI | Derivative of pUC19 (New England Biolabs) with *AclI-pTEF-AjuI.* |
| pUC19_AvrII_PrTEF_AjuI | Derivative of pUC19 (New England Biolabs) with *AvrII-pTEF-AjuI.* |
| pUC19_SdaI_PrTEFin(-) | Derivative of pUC19 (New England Biolabs) with *SdaI-pTEF* with the first intron of *tef* from *Y. lipolytica* followed by *SnaBI.* |
| pUC19_ApaLI_PrTEFin(-) | Derivative of pUC19 (New England Biolabs) with *ApaLI-pTEF* with the first intron of *tef* from *Y. lipolytica* followed by *SnaBI.* |
| pUC19_AclI_PrTEFin(-) | Derivative of pUC19 (New England Biolabs) with *AclI-pTEF* with the first intron of *tef* from *Y. lipolytica* followed by *SnaBI.* |
| pUC19_AvrII_PrTEFin(-) | Derivative of pUC19 (New England Biolabs) with *AvrII-pTEF* with the first intron of *tef* from *Y. lipolytica* followed by *SnaBI.* |
| pUC19_SdaI_hpNdin_cassette | Derivative of pUC19 (New England Biolabs) with *SdaI-minLEU2* with the first intron of *tef* from *Y. lipolytica* followed by *SnaBI.* |
| pUC19_ApaLI_hpNdin_cassette | Derivative of pUC19 (New England Biolabs) with *ApaLI-minLEU2* with the first intron of *tef* from *Y. lipolytica* followed by *SnaBI.* |
| pUC19_AclI_hpNdin_cassette | Derivative of pUC19 (New England Biolabs) with *AclI-minLEU2* with the first intron of *tef* from *Y. lipolytica* followed by *SnaBI.* |
| pUC19_AvrII_hpNdin_cassette | Derivative of pUC19 (New England Biolabs) with *AvrII-minLEU2* with the first intron of *tef* from *Y. lipolytica* followed by *SnaBI.* |
| pPrTEFAf4 | Derivative of pKG2-PIS with PC_T integrated into *SdaI* and *PacI* |
| pU4PrTEFAf4 | Derivative of pKG2-PIS with PC_U4T integrated into *SdaI* and *PacI* |
| pPrTEFinAf4 | Derivative of pKG2-PIS with PC_Ti integrated into *SdaI* and *PacI* |
| pU4PrTEFinAf4 | Derivative of pKG2-PIS with PC_U4Ti integrated into *SdaI* and *PacI* |
| pU16PrTEFinAf4 | Derivative of pKG2-PIS with PC_U16Ti integrated into *SdaI* and *PacI* |
| php4dAf4 | Derivative of pKG2-PIS with PC_U4M integrated into *SdaI* and *PacI* |
| php16dAf4 | Derivative of pKG2-PIS with PC_U16M integrated into *SdaI* and *PacI* |
| php4dinAf4 | Derivative of pKG2-PIS with PC_U4Mi integrated into *SdaI* and *PacI* |
| php16dinAf4 | Derivative of pKG2-PIS with PC_U16Mi integrated into *SdaI* and *PacI* |
| php4dAf4_1xL | Derivative of pKG2-PIS with PC_U4ML integrated into *SdaI* and *PacI* |
| php4dinAf4_1xL | Derivative of pKG2-PIS with PC_U4MiL integrated into *SdaI* and *PacI* |

Table S4: Assembly and sequencing primers. Overlaps are shown in bold, and restriction sites are underlined.

| **Type** | **Primer** | **Sequence (5‘🡪3‘)** |
| --- | --- | --- |
| Amplification | Pr_FW_pTEFin(-) | **AGGTCGACTCTAGAGGATC**CCCGGGAGAGACCGGGTTGGCGGCGCATTTG |
|  | Pr_RW_pTEFin(-) | **AGTGAATTCGAGCTCGG**TACGTACTGCAAAAAGTGCTGGTCGGAT |
|  | Pr_Ver_pUC19_MCS_FW | GGTTTCGCCACCTCTGACTTGAGC |
|  | Pr_Ver_pUC19_MCS_RW | GTGCCACCTGACGTCTAAGAAACC |
|  | Pr_Pfa1_FW_pTEFin | **ACCAGCACTTTTTGCAGTAC**TAACCGCAGTCCGCTATTGGCCGATGGAATGCTC |
|  | Pr_Lip2t_RW_ApaLI | **AGTGAATTCGAGCTCGGTAC**GTGCACGGTTTCGATTTGTCTTAGAGG |
|  | Pr_Pfa2_FW_pTEFin | **ACCAGCACTTTTTGCAGTAC**TAACCGCAGACCCAAGTCCCCGTTGCTATTGTC |
|  | Pr_Lip2t_RW_AclI | **AGTGAATTCGAGCTCGGTAC**AACGTTGGTTTCGATTTGTCTTAGAGG |
|  | Pr_Pfa3_FW_pTEFin | **ACCAGCACTTTTTGCAGTAC**TAACCGCAGACCTTTGAACCTATTGCTATCGTCG |
|  | Pr_Lip2t_RW_AvrII | **AGTGAATTCGAGCTCGGTAC**CCTAGGGGTTTCGATTTGTCTTAGAGG |
|  | Pr_Ppt_FW_pTEFin | **ACCAGCACTTTTTGCAGTAC**TAACCGCAGGCCCTGCTGGACCTGCCCCGAGGAG |
|  | Pr_Lip2t_RW_PacI | **AGTGAATTCGAGCTCGGTAC**TTAATTAAGGTTTCGATTTGTCTTAGAGG |
|  | Pr_Pfa1_FW_pTEF | **GAGTATAAGAATCATTCAAA**ATGTCCGCTATTGGCCGATGGAATGCTC |
|  | Pr_Lip2t_RW_ApaLI_pTEFgibson | **ACCCGAGGAGTGCGCGGATT**GTGCACGGTTTCGATTTGTCTTAGAGG |
|  | Pr_Pfa2_FW_pTEF | **GAGTATAAGAATCATTCAAA**ATGACCCAAGTCCCCGTTGCTATTGTC |
|  | Pr_Lip2t_RW_AclI_pTEFgibson | **ACCCGAGGAGTGCGCGGATT**AACGTTGGTTTCGATTTGTCTTAGAGG |
|  | Pr_Pfa3_FW_pTEF | **GAGTATAAGAATCATTCAAA**ATGACCTTTGAACCTATTGCTATCG |
|  | Pr_Lip2t_RW_AvrII_pTEFgibson | **ACCCGAGGAGTGCGCGGATT**CCTAGGGGTTTCGATTTGTCTTAGAGG |
|  | Pr_Ppt_FW_pTEF | **GAGTATAAGAATCATTCAAA**ATGGCCCTGCTGGACCTGCCCCGAGGAG |
|  | Pr_Lip2t_RW_PacI_pTEFgibson | **ACCCGAGGAGTGCGCGGATT**TTAATTAAGGTTTCGATTTGTCTTAGAGG |
|  | Pr_UAS1BN_Syn_FW | GTCGACTCTAGAGGATCCCC |
|  | Pr_UAS1BN_Syn_RW | CCGCCAACCCGGTCTCTCCC |
|  | Pr_pUC19_mutMCS_FW_v4 | **GCCAAGCTTGCATGCCTGCA**CCAATACGCAAACCGCCTCTCCC |
|  | Pr_pUC19_mutMCS_ApaLI_RW_v4 | **TCCTCTAGAGTCGACCTGCA**GTGCACGGGTGCCTAATGAGTGAGCTAAC |
|  | Pr_pUC19_mutMCS_AclI_RW_v4 | **TCCTCTAGAGTCGACCTGCA**AACGTTGGGTGCCTAATGAGTGAGCTAAC |
|  | Pr_pUC19_mutMCS_AvrII_RW_v4 | **TCCTCTAGAGTCGACCTGCA**CCTAGGGGGTGCCTAATGAGTGAGCTAAC |
|  | Pr_minLEU2_SdaI | CCTGCAGGGTCGACTCTAGAGGATCCCCGGGAGAGACCGGGTTGGCGGCATGCACTGATCACGGGCAAAAG |
|  | Pr_minLEU2_ApaLI | GTGCACGTCGACTCTAGAGGATCCCCGGGAGAGACCGGGTTGGCGGCATGCACTGATCACGGGCAAAAG |
|  | Pr_minLEU2_AclI | AACGTTGTCGACTCTAGAGGATCCCCGGGAGAGACCGGGTTGGCGGCATGCACTGATCACGGGCAAAAG |
|  | Pr_minLEU2_AvrII | CCTAGGGTCGACTCTAGAGGATCCCCGGGAGAGACCGGGTTGGCGGCATGCACTGATCACGGGCAAAAG |
|  | Pr_M13_pUC57_FW | CCCAGTCACGACGTTGTAAAACG |
|  | Pr_M13_pUC57_RW | AGCGGATAACAATTTCACACAGG |
|  | Pr_pTEF_AjuI_RW | TGAATTCGAGCTCGGTACCCGAGGAGTGCGCGGAACTGTTCCAAAATGCTTTTCTAAGTTG**TTTGAATGATTCTTATACTCAGAAGGAAATG** |
|  | Pr_Lip2t_RW_SdaI | **AGTGAATTCGAGCTCGGTAC**CCTGCAGGGGTTTCGATTTGTCTTAGAGG |
|  | Pr_Link1_FW_v2 | TCTAAGACAAATCGAAACCGGGATCCGGCGC |
|  | Pr_Link1_RW_v2 | GGGATCCTCTAGAGTCGACGGCGAAGACCTGTCGAGT |
|  | Pr_Link2_FW_v2 | CTAAGACAAATCGAAACCAAACGCCAGCAAGACGTAGC |
|  | Pr_Link2_RW_v2 | GGATCCTCTAGAGTCGACAAGGCAGCGCTCTGGGT |
|  | Pr_Link3_FW_v2 | TCTAAGACAAATCGAAACCCGGCACCTGTCCT |
|  | Pr_Link3_RW_v2 | GGGATCCTCTAGAGTCGACCTTCCTTGCGGCGGCG |
| Sequencing | PR_seq_Pfa1_reverse | GCTGGCTGACAAATACGACTACAC |
|  | PR_seq_Pfa1_1_forward | GGGTCGAGGTCGGAAATCAAAG |
|  | PR_seq_Pfa1_2_forward | GTTGTCGGTATGACCACCAG |
|  | PR_seq_Pfa2_reverse | ACTTTGGCGTTTCCGTTTCC |
|  | PR_seq_Pfa2_1_forward | GACCTCAGCAGGTCGAACAATG |
|  | PR_seq_Pfa2_2_forward | GATCCTTCGATCTCAGCGATG |
|  | PR_seq_Pfa2_3_forward | AAGCCACAACCACAGAGGATCGAG |
|  | PR_seq_Pfa2_4_forward | ATTTCAGAGGCCACCACCTCAG |
|  | PR_seq_Pfa2_5_forward | CTTAATAGAGTCGATGCCCAGGTC |
|  | PR_seq_Pfa2_6_forward | CGTGCTTTCCCTTTCGATCC |
|  | PR_seq_Pfa2_7_forward | GGTAGAAGAGGCGGCATTCATC |
|  | PR_seq_Pfa2_8_forward | GGCATCAGAGGCAACGATAG |
|  | PR_seq_Pfa2_9_forward | CAGCTTCAGTCCGCCAAATTC |
|  | PR_seq_Pfa2_10_forward | CGTTAGTGATTCGGTCACAGATTC |
|  | PR_seq_Pfa3_reverse | TTTATTATGGTCGCTTCTGCC |
|  | PR_seq_Pfa3_1_forward | GAATTCTCCCAGCCATCGATCC |
|  | PR_seq_Pfa3_2_forward | GACCTGGTGGTTAGCGACATAGAG |
|  | PR_seq_Pfa3_3_forward | GCAGTCCGACCTGATTGTCAAAG |
|  | PR_seq_Pfa3_4_forward | ATCGACCTTAACAGAAGGAATAGG |
|  | PR_seq_Pfa3_5_forward | AGGAGTCGGTAGACTCGTTC |
|  | PR_seq_Pfa3_6_forward | ACGTGAGTGAACACAGGTTCGG |
|  | PR_seq_Pfa3_7_forward | TCAGCAGTGTTAATAATGGTCAGG |
|  | PR_seq_Pfa3_8_forward | TCCGACCCAAGGCATAGAAG |
|  | PR_seq_Pfa3_9_forward | CAGGGTTTGCTCCAGATCTC |
|  | PR_seq_Pfa3_10_forward | CTGGAGGGCAAAGAACACAG |
|  | LIP2t_fwd | GCGTTCCTCTAAGACAAATCGAAACC |
|  | Pr_PIS_genome_fwd | AATTCCCAGAGGTGTCGAGTGGC |
|  | Pr_PIS_genome_rev | TGTTCGCTTCTCCTGTCTACATTGG |
|  | Pr_seq_Ppt_fwd | CCGGTCTCCGATTTAACCTG |
|  | Pr_seq_Ppt_rev | GGGAGAGAGCTGGAAAGAGAATTG |
|  | Pr_seq_Prom_Pfa1 | CGCACGACGTGCAGAGATTC |
|  | Pr_seq_Prom_Pfa2 | TCTCGGGAAGCCACAATATC |
|  | Pr_seq_Prom_Pfa3 | GGAGAGGTCACGGCAGATTC |
| qRT PCR | Pr_qRT_Pfa1_FW | TGATGAGGGAAAGCGAATGC |
|  | Pr_qRT_Pfa1_RW | ACGCCGAGCTCAAACATATC |
|  | Pr_qRT_Pfa2_FW | TCTTTTGGTTTCGGCGGTTC |
|  | Pr_qRT_Pfa2_RW | AAGGGGCGGAAATCACAAAC |
|  | Pr_qRT_Pfa3_FW | TTTCTTGGCTGGGCATTGAC |
|  | Pr_qRT_Pfa3_RW | TCAGCATGTCCGTCAATGTG |
|  | Pr_qRT_PPt_FW_v2 | ACGACGACCTCGATTTTTCG |
|  | Pr_qRT_PPt_RW_v2 | TCCAGAACGGGATCAAAGGC |
|  | Pr_qRT_rRNA_FW | TAACACCTCGATGTCGGCTTAC |
|  | Pr_qRT_rRNA_RW | ACCGTGCTATCTCACAATGC |

Table S5. Generated genetic building blocks for cluster assembly

| **Type** | **Building block** | **Description** |
| --- | --- | --- |
| **UAS** | BB_U4 | UAS1B4 (U4) |
|  | BB_U16 | UAS1B16 (U16) |
|  | BB_S1 | Spacer1 |
| **Spacer** | BB_S2 | Spacer2 |
|  | BB_S3 | Spacer3 |
| **SGUs** | BB_T1 | *SdaI*-pTEF-*pfa1-ApaLI* |
|  | BB_U4T1 | *SdaI*-U4-pTEF-*pfa1-Lip2t-ApaLI* |
|  | BB_T2 | *ApaLI*-pTEF-*pfa2-AclI* |
|  | BB_U4T2 | *ApaLI*-U4-pTEF-*pfa2-AclI* |
|  | BB_T3 | *AclI*-pTEF-*pfa3-AvrII* |
|  | BB_U4T3 | *AclI*-U4-pTEF-*pfa3-AvrII* |
|  | BB_TP | *AvrII*-pTEF-*ppt-PacI* |
|  | BB_U4TP | *AvrII*-U4-pTEF-*ppt-PacI* |
|  | BB_Ti1 | *SdaI*-pTEF-Int-*pfa1-ApaLI* |
|  | BB_U4Ti1 | *SdaI*-U4-pTEF-Int-*pfa1-ApaLI* |
|  | BB_U16Ti1 | *SdaI*-U16-pTEF-Int-*pfa1-ApaLI* |
|  | BB_Ti2 | *ApaLI*-pTEF-Int-*pfa2-AclI* |
|  | BB_U4Ti2 | *ApaLI*-U4-pTEF-Int-*pfa2-AclI* |
|  | BB_U16Ti2 | *ApaLI*-U16-pTEF-Int-*pfa2-AclI* |
|  | BB_Ti3 | *AclI*-pTEF-Int-*pfa3-AvrII* |
|  | BB_U4Ti3 | *AclI*-U4-pTEF-Int-*pfa3-AvrII* |
|  | BB_U16Ti3 | *AclI*-U16-pTEF-Int-*pfa3-AvrII* |
|  | BB_TinP | *AvrII*-pTEF-Int-*ppt-PacI* |
|  | BB_U4TiP | *AvrII*-U4-pTEF-Int-*ppt-PacI* |
|  | BB_U16TiP | *AvrII*-U16-pTEF-Int-*ppt-PacI* |
|  | BB_U4M1 | *SdaI*-U4-pminLEU2-*pfa1-ApaLI* |
|  | BB_U16M1 | *SdaI*-U16-pminLEU2-*pfa1-ApaLI* |
|  | BB_U4M2 | *ApaLI*-U4-pminLEU2-*pfa2-AclI* |
|  | BB_U16M2 | *ApaLI*-U16-pminLEU2-*pfa2-AclI* |
|  | BB_U4M3 | *AclI*-U4-pminLEU2-*pfa3-AvrII* |
|  | BB_U16M3 | *AclI*-U16-pminLEU2-*pfa3-AvrII* |
|  | BB_U4MP | *AvrII*-U4-pminLEU2-*ppt-PacI* |
|  | BB_U16MP | *AvrII*-U16-pminLEU2-*ppt-PacI* |
|  | BB_U4Mi1 | *SdaI*-U4-pminLEU2-Int-*pfa1-ApaLI* |
|  | BB_U16Mi1 | *SdaI*-U16-pminLEU2-Int-*pfa1-ApaLI* |
|  | BB_U4Mi2 | *ApaLI*-U4-pminLEU2-Int-*pfa2-AclI* |
|  | BB_U16Mi2 | *ApaLI*-U16-pminLEU2-Int-*pfa3-AclI* |
|  | BB_U4Mi3 | *AclI*-U4-pminLEU2-Int-*pfa3-AvrII* |
|  | BB_U16Mi3 | *AclI*-U16-pminLEU2-Int-*pfa3-AvrII* |
|  | BB_U4MiP | *AvrII*-U4-pminLEU2-Int-*ppt-PacI* |
|  | BB_U16MiP | *AvrII*-U16-pminLEU2-Int-*ppt-PacI* |

Table S6: Generated PUFA clusters. BB: building blocks; PC: PUFA cluster.

| **Cluster variants** | **Description** |
| --- | --- |
| PC_T | BB_T1 + BB_T2 + BB_T3 + BB_TP |
| PC_U4T | BB_U4T1 + BB_U4T2 + BB_U4T3 + BB_U4TP |
| PC_Ti | BB_Ti1 + BB_Ti2 + BB_Ti3 + BB_TiP |
| PC_U4Ti | BB_U4Ti1 + BB_U4Ti2 + BB_U4Ti3 + BB_U4TiP |
| PC_U16Ti | BB_U16Ti1 + BB_U16Ti2 + BB_U16Ti3 + BB_U16TiP |
| PC_U4M | BB_U4M1 + BB_U4M2 + BB_U4M3 + BB_U4MP |
| PC_U16M | BB_U16M1 + BB_U16M2 + BB_U16M3 + BB_U16MP |
| PC_U4Mi | BB_U4Mi1 + BB_U4Mi2 + BB_U4Mi3 + BB_U4MiP |
| PC_U16Mi | BB_U16Mi1 + BB_U16Mi2 + BB_U16Mi3 + BB_U16MiP |
| PC_U4ML | BB_U4M1 + BB_S1 + BB_U4M2 + BB_S2 + BB_U4M3 + BB_S3 + BB_U4MP |
| PC_U4MiL | BB_U4Mi1 + BB_S1 + BB_U4Mi2 + BB_S2 + BB_U4Mi3 + BB_S3 + BB_U4MiP |
